# Photolytic radical persistence due to anoxia in viscous aerosol particles

**DOI:** 10.1038/s41467-021-21913-x

**Published:** 2021-03-19

**Authors:** Peter A. Alpert, Jing Dou, Pablo Corral Arroyo, Frederic Schneider, Jacinta Xto, Beiping Luo, Thomas Peter, Thomas Huthwelker, Camelia N. Borca, Katja D. Henzler, Thomas Schaefer, Hartmut Herrmann, Jörg Raabe, Benjamin Watts, Ulrich K. Krieger, Markus Ammann

**Affiliations:** 1 Laboratory of Environmental Chemistry, Paul Scherrer Institute, Villigen, Switzerland; 2 Institute for Atmospheric and Climate Science, ETH Zurich, Zurich, Switzerland; 3 Laboratory for Synchrotron Radiation and Femtochemistry, Paul Scherrer Institute, Villigen, Switzerland; 4 Atmospheric Chemistry Department, Leibniz Institute for Tropospheric Research, Leipzig, Germany; 5 Laboratory for Synchrotron Radiation-Condensed Matter, Paul Scherrer Institute, Villigen, Switzerland; 6 Present Address: Laboratory for Physical Chemistry, ETH Zurich, Zurich, Switzerland

**Keywords:** Atmospheric chemistry, Atmospheric chemistry, Photochemistry, Chemical physics

## Abstract

In viscous, organic-rich aerosol particles containing iron, sunlight may induce anoxic conditions that stabilize reactive oxygen species (ROS) and carbon-centered radicals (CCRs). In laboratory experiments, we show mass loss, iron oxidation and radical formation and release from photoactive organic particles containing iron. Our results reveal a range of temperature and relative humidity, including ambient conditions, that control ROS build up and CCR persistence in photochemically active, viscous organic particles. We find that radicals can attain high concentrations, altering aerosol chemistry and exacerbating health hazards of aerosol exposure. Our physicochemical kinetic model confirmed these results, implying that oxygen does not penetrate such particles due to the combined effects of fast reaction and slow diffusion near the particle surface, allowing photochemically-produced radicals to be effectively trapped in an anoxic organic matrix.

## Abstract (web summary)

Sunlight can change the composition of atmospheric aerosol particles, but the mechanisms through which this happens are not well known. Here, the authors show that fast radical reaction and slow diffusion near viscous organic particle surfaces can cause oxygen depletion, radical trapping and humidity dependent oxidation.

## Introduction

Aerosol particles suspended in the atmosphere can take up water and initiate the formation of liquid or ice clouds, impact Earth’s radiative balance^1,2^, and cause oxidative stress when inhaled affecting respiratory health^3,4^. Atmospheric aerosol components that generate radicals and reactive oxygen species (ROS) in lungs are linked to toxicity, inflammation, lung disease, and thus, loss of human life^4–6^. Species of ROS include the hydroxyl radical, OH, the hydroperoxyl radical, HO_2_, the superoxide radical anion, ${\mathrm{O}}_2^ -$, and hydrogen peroxide, H_2_O_2_, among others. Of particular interest are carbon-centered radicals (CCRs), such as the methyl radical, ^•^CH_3_, or the triphenylmethyl radical, ^•^C(C_6_H_5_)_3_, with very different lifetimes. They are some of the many types of radicals that are present in aerosol particles produced from combustion sources, like biomass burning or cigarette smoke; photochemical reactions and aqueous aerosol chemistry; ozonolysis of certain biogenic or anthropogenic gaseous compounds; and biogenic aerosol sources, such as pollen proteins^4–8^. The lifetimes of ROS and CCRs are typically short due to their high reactivity. Yet, some CCRs can have a lifetime on the order of a day, termed environmentally persistent free radicals (EPFR), which are stabilized with the help of aromatic species or by forming organo-metal complexes in particles or on surfaces^5–7^.

In cold and dry air, aqueous aerosol particles that are dominated by organic solutes may attain a high viscosity^9,10^ and become solid-like, which slows molecular transport and thus radical chemistry^11–13^. Organic matter is abundant in atmospheric particles and frequently mixes with inorganic and trace metal compounds^3,4,6,8,14,15^. In atmospheric particles, iron can undergo rapid photochemical cycling leading to radical formation and driving chemical change^7,8,16–21^. Photochemical reactions in aerosol particles occur throughout their bulk and produce CCRs, but we hypothesize that subsequent processes, such as ROS production, evaporation, or gas phase O_2_ uptake and reaction with CCRs can be significantly stunted by molecular diffusion limitations. Iron(III)-citrate (Fe^III^Cit) photochemistry is representative of iron-carboxylate photochemistry^22^, which is thought to be a dominant source of OH radicals in atmospheric aerosol particles and the dominant sink of carboxylic acids^18,23^. Fe^III^Cit undergoes ligand to metal charge transfer (LMCT), iron reduction, and decarboxylation in the same way as many other atmospherically important photoactive iron(III) carboxylate compounds^18,23,24^. Citric acid is also a well-known proxy for atmospheric organic aerosol particles in terms of composition and viscosity^25^. Photochemically generated radicals that build up and persist may affect lifetimes of other aerosol-bound species such as pathogens or toxins, with health-related impacts.

Here, we show that photochemically produced CCRs are immobilized in highly viscous organic aerosol particles and act as a major sink for O_2_ inside particles, thereby inducing anoxia. Atmospheric aerosol anoxia is highly unexpected since the time it takes for small molecules, such as water and possibly oxygen, to diffuse and mix throughout non-reactive viscous particles is thought to be on the order of seconds to minutes at ambient conditions^11,13,26,27^. However, we found that it is the combined effects of reaction and diffusion limitations that ROS and CCRs can persist and accumulate in viscous aerosol particles and completely overwhelm O_2_ in their interior. This occurs under both cold and warm conditions, but largely depending on the relative humidity, RH, particles are exposed to. Persistent ROS and CCR species can then be released under more humid conditions, e.g., upon inhalation, which will exacerbate the negative health effects of aerosol exposure beyond what is expected when assuming a well-mixed and a highly reactive aerosol matrix during their airborne trajectory.

## Results

### Looking inside single particles for their chemical morphology

**Fig. 1:**
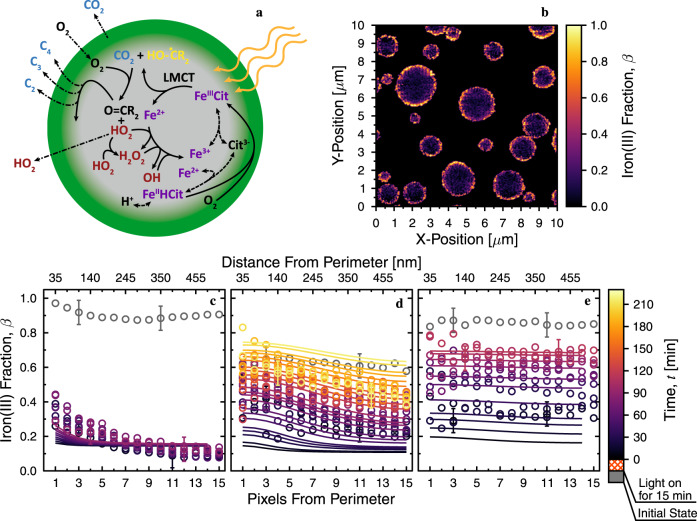
Imaged photochemical and oxidative cycling of iron(III)-citrate (Fe^III^Cit) inside single aerosol particles. **a** Sketch of Fe^III^Cit photochemical cycling in a particle oxidized near its surface (green) and anoxic in its bulk (gray). Purple, blue, and red text indicate specific species discernable using scanning transmission X-ray microscopy coupled to near-edge X-ray absorption fine structure spectroscopy (STXM/NEXAFS), an electrodynamic balance (EDB) and coated wall flow tube (CWFT) methods, respectively. Solid lines are chemical reactions, e.g., ligand-to-metal charge transfer (LMCT) reaction, dashed lines are equilibria conditions, and dash-dotted lines indicate gas evaporation or uptake. Citrate, the iron(II)-citrate complex, and CH_2_COOH are abbreviated as Cit^3-^, Fe^II^HCit, and R, respectively. **b** A STXM/NEXAFS image of the iron(III) fraction, *β*, in particles directly showing reoxidation only near the surface of particles. The image was smoothed by increasing the pixel resolution and interpolating. **c**–**e** Chemical gradients inside particles as a function of time, *t* (color coded), and relative humidity, RH, during dark reoxidation at 20 °C after 15 min of UV light exposure. At RH = 40% (**c**), dark reoxidation occurred slowly while maintaining gradients in *β*. Reoxidation at RH = 50% (**d**) and 60% (**e**) was faster, implying that particles took up more water resulting in greater diffusion coefficients, hence faster molecular transport and oxidation reaction. Gray symbols are the measured initial iron(III) fraction, *β*_0_, from multiple images acquired before UV light exposure. Example error bars, Δ*β* = ±0.07, are shown. Solid colored lines are derived from the photochemical reaction and diffusion (PRAD) model and reproduce our observations. Details on model sensitivity are given in the Supplementary Information (Supplementary Fig. 3).

The Fe^III^Cit photochemically reactive system shown in Fig. 1a was employed to produce CCRs in the presence of O_2_, the gas phase reactant of interest. We used a triad of photochemical experiments to observe photochemical reaction cycles on the nanoscale and microscale in single particles and films including scanning transmission X-ray microscopy coupled with near-edge X-ray absorption fine structure (STXM/NEXAFS) spectroscopy, a single particle electrodynamic balance (EDB), and a coated-wall flow tube (CWFT). Our multifarious observations were supported by a recent photochemical reaction and diffusion (PRAD) model^28,29^. We observed that photochemically produced CCRs induce anoxia in the interior of viscous particles, effectively preserving CCRs under dry conditions. Figure 1 shows viscous Fe^III^Cit particles during photochemical cycling, where an oxidized shell and unoxidized core is depicted by the color gradient (Fig. 1a). This oxidation configuration was directly observed using STXM/NEXAFS to image iron(III) fraction, *β*, acquired minutes after photoreduction and reoxidation (Fig. 1b). These low *β* values are indicative of LMCT, i.e., charge transfer from the citrate ligand to Fe^3+^, forming Fe^2+^ and a primary radical product, which immediately decays by decarboxylation (i.e., the removal of a carboxyl group via loss of CO_2_). Then, an organic, carbon-centered radical, the primary CCR, is left behind^28^. Following the reaction steps in Fig. 1a, ROS is generated when O_2_ is taken up by the particle and reacts with photochemically generated CCRs, through mostly a first generation of peroxy radicals. Finally, iron(II) can be reoxidized by ROS or O_2_, then complexed again with citric acid. Low values of *β* observed within particles also imply an absence of the O_2_ and ROS needed for oxidation and thus a persistence of CCRs. We observed 2-D column integrated profiles (i.e., in the same dimension as the 2-D microscope image) of *β* in aerosol particles evolving in the dark at RH = 40, 50, and 60%, Fig. 1c–e respectively, to discover where oxidation occurred inside single particles. At RH = 40%, *β* remained low and indicated that CCRs remained at high concentrations and unreacted. Gradients in *β* were observed at all RH, but most discernable for RH ≤ 50% and extended throughout the interior of particles. At RH = 60%, profiles of *β* were relatively flat from the perimeter to the center of particles (Fig. 1e). This indicates a transition from an inhomogeneous state, characterized by anoxic conditions at RH = 40%, to homogeneous, well-mixed conditions enhancing O_2_ concentrations in the bulk. Therefore, we conclude that altering RH significantly alters the diffusion coefficients of reactants and products, and so controls whether or not radicals are built up or reacted away quickly.

X-ray imaging shows that oxidation occurred near particle surfaces, but not in their bulk. Therefore, photochemically generated CCRs present in the bulk must have had long lifetimes due to slow molecular transport and reaction with O_2_ as their main reaction partner. Additional images that support this finding acquired at the start and end of STXM/NEXAFS experiments are given in Supplementary Fig. 1. We used the PRAD model^28^ to simulate radical persistence due to photochemical reduction and dark reoxidation experiments performed with STXM/NEXAFS (solid lines in Fig. 1c–e). Modeled profiles of *β* reproduce our observations only when the modeled O_2_ concentration drops to zero just nanometers beneath the surface of an aerosol particle as shown in the radial profiles in Supplementary Fig. 2. The accumulation of CCRs in anoxic particle interiors is the necessary condition to explain the spectroscopically observed iron oxidation state, pointing to long radical lifetimes in photoactive, viscous aerosol particles. When particles with high radical concentration are inhaled, they may release CCRs and ROS in the respiratory tract. It is conceivable that the saturated lung environment lets non-organic and iron species undergo chemical cycling to produce even more ROS and CCRs, exacerbating lung tissue damage.

### Photochemical evolution of particle mass loss and radical release

**Fig. 2:**
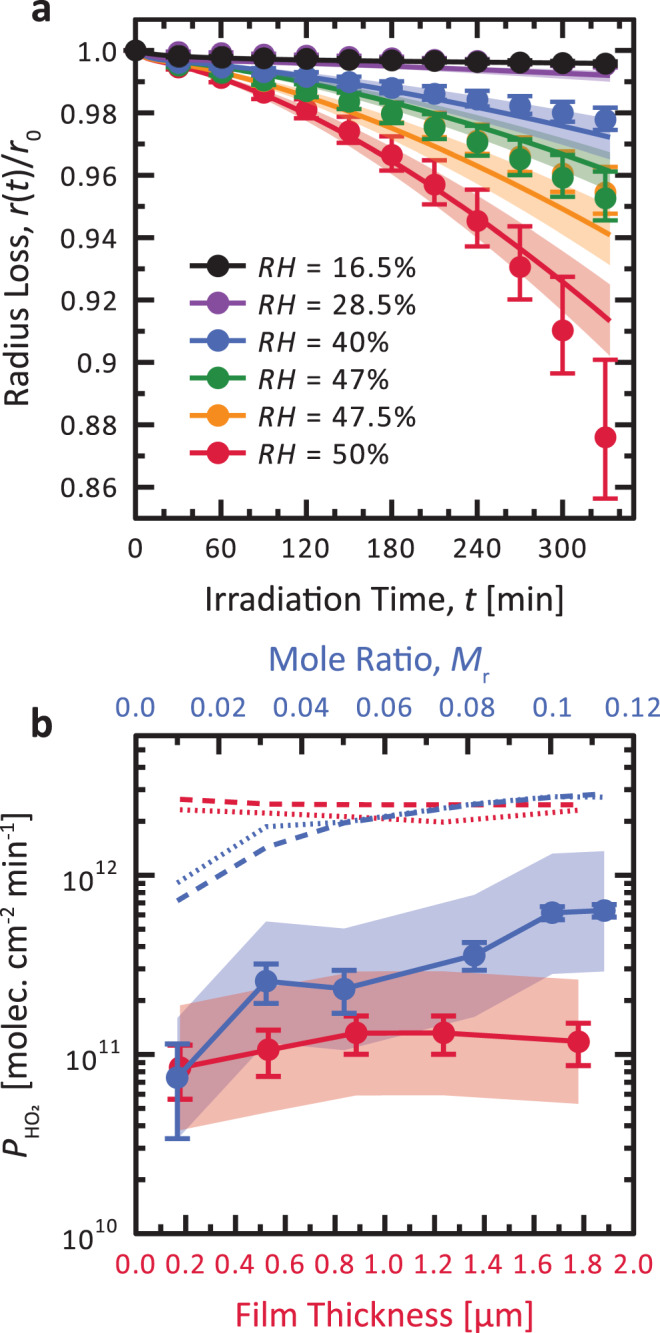
Photochemical reaction in particles and films. **a** Electrodynamic balance experiments determined that particle radius, *r*(*t*), decreased over time, *t*, during light exposure (*r*_0_ = *r*(0)) due to the release of CO_2_ and other volatile species. Error bars indicate the range of possible refractive index values from any changes in composition during irradiation. The solid lines were derived from the photochemical reaction and diffusion (PRAD) model, and the shading is the model sensitivity to ±2% in relative humidity, RH. **b** Measured steady-state production of HO_2_, $P_{{\mathrm{HO}}_2}$, from coated wall flow tube experiments is shown as a function of the mole ratio between Fe(III)-citrate and citric acid, *M*_r_ (blue), and film thickness (red) indicated by symbols. Error bars indicate the standard deviation of multiple measurements or the propagated random error from solution preparation, whichever is larger. Dotted and dashed lines were determined from the PRAD model with *k*_SR_ = 8.3 × 10^5^ M^−1^ s^−1^ in dilute aqueous solution, or using the parameterization $$\begin{array}{l}\log k_{{\mathrm{SR}}} = - 2.854 \times 10^{ - 5}{\mathrm{RH}}^3 + 0.0024{\mathrm{RH}}^2 + 0.1087{\mathrm{RH}} - 0.05018,\end{array}$$ respectively^28^. Here, M is molarity in units of mole L^−1^. Solid lines were from the PRAD model using fitted HO_2_ loss rates shown in Supplementary Fig. 6. The shading is the model sensitivity to a factor of 5 uncertainty in the HO_2_ loss rate.

We irradiated single particles, which were levitated in an EDB, with visible light for more than 5 h (Fig. 2a) and observed their continual size decrease. Under humid conditions (RH = 50%), a 1 µm decrease in particle radius, *r*, was observed (symbols), i.e., about 12% of its initial value, *r*_0_ = 8 µm. This is equivalent to a ∼33% mass loss. In contrast, at RH = 16%, irradiation caused only a 30 nm decrease in *r*, i.e., about ∼0.4% in *r*. This small mass loss was due to the fact that ROS and CCR, though continuously produced, did not produce and release significant amount of CO_2_ or volatile compounds to the gas phase. In general, EDB observations are master constraints for modeling radical chemistry occurring in the condensed phase of citric acid initiated by photolysis of iron carboxylate complexes^28^. All modeled radii decrease (solid lines) for all RH shown in Fig. 2a, which is in agreement with our data. The decrease in *r* accelerated, as seen in Fig. 2a, which is likely the result of CO_2_ release and volatile product formation (indicated as C_2–5_ in Fig. 1a) and their increasing evaporation at later times. We note that accelerated decrease in *r* may be counter to a competitive CCR recombination reaction, in which CCRs would destroy themselves, leading to less volatile, higher molecular weight products such as oligomers^30^. We have found evidence shown in Supplementary Fig. 4 that CCR recombination may occur on long time scales, which is likely not significant enough to affect the observed fast photochemical cycling resulting in the loss of mass. Although radical reactions are typically fast in dilute solution^31^, they can be limited due to diffusion-controlled reaction^32^. In conjunction, CCRs may also become stabilized with iron^6,7^, in line with our hypothesis that they persist in anoxic viscous organic particles (see Supplementary Fig. 4). Unimolecular radical reactions are known to eliminate CO_2_ and HO_2_, as observed here^16,18,22–24,31,33–35^. In other cases, elimination reactions may result in producing new radicals, and not their overall destruction (e.g., H_2_O elimination followed by glycol radical production)^36^. We speculate that an elimination reaction for CCRs would likely contribute to the production of CO_2_, VOCs, and small radical compounds, and therefore propagate radical persistence and continued radical chemistry. The PRAD model reproduces our results when CCR self-reaction is turned off, indicating that ROS and CCR turnover is important near the surface of particles where oxidation takes place, and that the lifetime of CCRs must be long in the cores of the particles.

Finally, the CWFT experiments shown in Fig. 2b show a steady-state production and release of HO_2_ radicals, $P_{{\mathrm{HO}}_2}$, demonstrating continual ROS production from photochemical cycling of Fe^III^Cit. This radical source was due entirely to O_2_ uptake and reaction. Increasing the Fe^III^Cit to citric acid mole ratio, *M*_r_, led to an increase in $P_{{\mathrm{HO}}_2}$, which was expected since a greater number of photochemically active reactants should increase ROS and CCR. At our lowest investigated *M*_r_ = 0.01, the PRAD model still predicts anoxic conditions and CCR persistence as seen in Supplementary Fig. 5. We also show a typical time-resolved radial profile of HO_2_ concentration in Supplementary Fig. 5c, where the maximum occurs when CCR and O_2_ concentration within particles drops significantly. We also observed that $P_{{\mathrm{HO}}_2}$ increased by a factor of 1.5 for film thicknesses 0.18–0.89 µm; however, this is far less than our error estimate from the HO_2_ loss rate. If there were a large gradient in O_2_ concentration over this range, we would expect $P_{{\mathrm{HO}}_2}$ would significantly depend on film thickness. As this was not the case, O_2_ penetration must have been much smaller than 0.18 µm, in agreement with STXM and EDB observations and modeling results. Recent ambient observations of soluble iron and ligand concentration in aerosol particles^20,21^ discussed later have shown that the mole ratio between iron organic complexes and organic compounds can be on the order of *M*_r_ = 10^−1^, which is consistent with the range in our CWFT and EDB experiments.

The observation of steady-state $P_{{\mathrm{HO}}_2}$ was the key observable of ensuing peroxy radical chemistry in the condensed phase. The PRAD model over predicted $P_{{\mathrm{HO}}_2}$ when using a HO_2_ self-reaction rate coefficient, *k*_SR_ = 8.3 × 10^5^ M^−1^ s^−1^, from dilute aqueous solution or using a previous parameterization by Dou et al.^28^ as a function of RH. Here, M is molarity in units of mole L^−1^. It appears that the condensed phase HO_2_ sink was greater than only considering its self-reaction and iron reoxidation by roughly 1 order of magnitude, indicating a central role of the organic peroxy radicals in the photochemical cycling, which are presently not known and not included in detail in the PRAD model. The PRAD model captured observed $P_{{\mathrm{HO}}_2}$ only when fitting the HO_2_ loss rate as described in the Supplementary Discussion (see Supplementary Fig. 6). Measurements of $P_{{\mathrm{HO}}_2}$ in dependence of film thickness and O_2_ partial pressure (Supplementary Fig. 6), measurements as a function of RH from Dou et al.^28^, and PRAD model results all allowed to confine primary peroxy radical formation to the outermost surface where O_2_ was still present. This reveals that H_2_O_2_ and likely second-generation peroxy radicals penetrated over depths larger than expected from its self-reaction. Supplementary Fig. 7 shows that H_2_O_2_, in particular, is a large contributor to the overall ROS concentration in the PRAD model. This is because the 1^st^ order chemical loss rate of H_2_O_2_ is slow (on the order of 10^−4^ s^−1^) and its solubility is high. Therefore, it has a high concentration in the condensed phase and enough time to diffuse through the particle. Calculated depth profiles of HO_2_ concentrations in films are shown in Supplementary Figs. 5 and 7 along with organic CCR, ROS, and O_2_ concentrations. Again, the PRAD model predicts complete O_2_ depletion at a short depth beneath the surface. Despite this limitation, any peroxy radical or hydroperoxides would contribute to the total ROS concentration, giving confidence to our quantification of ROS production for reoxidation and photochemical cycling. As mentioned above, HO_2_ peaked where O_2_ and CCRs were depleted, highlighting the importance of the reaction between them. In addition to EDB and STXM/NEXAFS experiments with high CCR concentrations, CWFT experiments further indicate that ROS existed at high concentrations, apparently replenished by the accelerating photochemical cycling.

## Discussion

The importance of oxygen limitation in photoactive particles is underscored by comparing environmentally persistent free radical concentrations [EPFR] in ambient air with our predictions. A previous study observed [EPFR] = 10^10^–10^12^ (molecules) μg^−1^ of aerosol mass, attributable to the presence of quinones and transition metals^5^. In highly viscous Fe^III^Cit and citric acid particles (RH = 20%), the ROS concentration, [ROS] = 2.7 × 10^10^ μg^−1^ and the CCR concentration was [CCR] = 1.5 × 10^12^ μg^−1^ at a quasi-steady state (Supplementary Fig. 7c). This emphasizes the importance of a persistent radical source from photochemistry, which is comparable to that of quinone and metal aerosol components. Compared to the range of values reported by ambient measurements, radical concentrations are not highly dependent on particle diameter between 0.03–1.0 µm (Supplementary Fig. 8). One caveat to predicting radical concentrations here is that we do not consider additional factors such as different morphologies, chemical inhomogeneity, or other competing atmospheric processes^15^, which may systematically enhance or hinder ROS and CCR buildup. In highly viscous particles, radicals are immediately produced and maintained over the course of hours as seen in Supplementary Fig. 7c. In lower viscosity particles, radicals are immediately produced, but subsequently drop by orders of magnitude. This implies that aerosol microphysics, and thus molecular diffusion, can change the progression of reactions tending toward drastically different outcomes in radical concentration. Previous measurements used electron paramagnetic resonance (EPR) spectroscopy on ambient particles collected on filters, which were then submerged in dilute aqueous solutions mimicking endogenous radical production in lungs and containing a radical trapping compound to quantify them^5^. Prior filter storage will probably lead to the loss of radicals exogenously produced due to aerosol photochemistry and thus would not be included in EPR analysis. Considering persistent radicals in aerosol particles due to photochemical processing may result in larger radical concentration compared to what is measured with filter methods or offline analysis. Therefore, there is a high risk to underestimate radical numbers in aerosol particle populations^37,38^ affecting a variety of consequences including the adverse health effects these particles cause^3,4,6–8,14^.

**Fig. 3:**
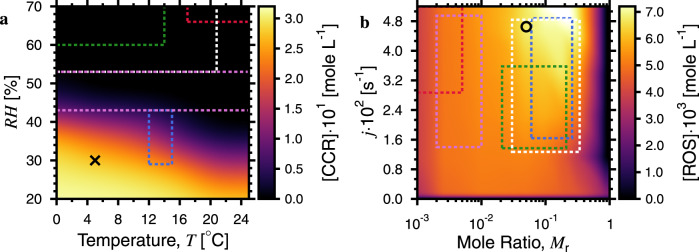
Microphysical control on predicting quasi-steady state carbon centered radicals (CCR) and reactive oxygen species (ROS) concentration in the atmosphere. **a** CCR concentration, [CCR], as a function of temperature, *T*, and relative humidity, *RH*, in particles is shown. High [CCR] occurred primarily at drier conditions and lower *T* to a lesser degree. **b** ROS concentration, [ROS], as a function of the mole ratio between iron(III)-citrate (Fe^III^Cit) and citric acid, *M*_r_, and the photochemical reaction rate, *j*. The maximum ordinate value, *j* = 5.23 × 10^-2^ s^-1^, corresponds to the photochemical dissociation rate for Fe^III^Cit with a light intensity at the Earth’s surface at 0° zenith. *M*_r_ and *j* values in **a** are indicated by the circle in **b**. *T* and RH values in **b** are indicated by the cross in **a**. [CCR] and [ROS] were averaged over the last 30 min for a particle having a radius of 0.5 µm in a 1 h simulation using the photochemical reaction and diffusion (PRAD) model. Dashed colored rectangles indicate the range of values for various field studies held in the southern Great Plains, USA^17^ (purple), Aksu, China^19^ (blue), urban centers in Canada^20^ (white), the Po Valley of Italy^21^ (green), and Okinawa Island^39^ (red) detailed in the Supplementary Information.

The range of atmospheric conditions under which ROS and CCRs can persist in aerosol populations is shown in Fig. 3 as a function of temperature, *T*, RH, *M*_r_, and the photochemical reaction rate, *j*. Conditions at which RH = 40–50% over *T* = 0–25 °C marks a transition between CCR buildup and depletion in aerosol particles entirely due to changing diffusion coefficients. Trends in [ROS] and [CCR] were similar as seen in Supplementary Fig. 9. At constant *T* and RH, [ROS] increased with *M*_r_ and reached a maximum of about 7 mM between *M*_r_ = 0.04–0.3. Inhaled particles with this level of exogenously produced ROS may contribute significantly to the total production in lungs (exogenous and endogenous), which is discussed in more detail in the Supplementary Information. When *M*_r_ increased further, [ROS] sharply decreased as seen in Fig. 3b. This was because O_2_ loss from CCR reaction became so extreme that O_2_ penetration, and thus ROS production became limited to <0.3 nm beneath the surface, i.e., the molecular scale. Additionally, an excess of Fe^2+^ resulted in a short ROS lifetime. In the PRAD model, diffusion coefficients for *M*_r_ > 0.05 decrease, which is evident from measured viscosities at *M*_r_ = 1.0 and 0.05 (Supplementary Fig. 11), contributing to this sub-nanometer penetration depth. Overall, we argue that ROS and CCR build-up in aerosol particles at the Earth’s surface are controlled, in part, by meteorological conditions and aerosol composition.

The rectangles in Fig. 3 allow comparing our estimates of ROS and CCR build-up with those previous field studies^17,19–21,39^, which detail aerosol composition, and specifically iron concentration, solubility, or speciation. We estimated *M*_r_ from previous studies using measured aerosol composition and 3 conservative assumptions, (i) that a fraction of iron in particles is soluble, (ii) that a fraction of soluble iron is in complex with carboxylate compounds, and (iii) that only a fraction of total particles (by number) contain iron. More details on estimates of *M*_r_ are in the Supplementary Information and Supplementary Table 2. In more continental and drier climates, as was the case for field studies in central China^19^ and the Great Plains of the USA^17^ (shown as the blue and purple boxes), *T* and RH were sufficiently low on average to promote radical buildup. In other field studies in Okinawa Island^39^, multiple urban sites in Canada^20^ and in the Po Valley of Italy^21^, climates are more humid on average. This implies that high [CCR] and [ROS] should occur across the Earth where aerosol particles are more viscous with long mean equilibrium mixing times, as previously reported^40^. Even under warm or humid average meteorological conditions, cold or dry weather spells can still lead to episodes of high radical concentrations. In the Po Valley of Italy^21^, metal concentrations were similar to other European urban areas with abundant iron carboxylate complexes (green box in Fig. 3). Therefore, we argue that iron carboxylate complexes are an ubiquitous aerosol component, in line with previous studies quantifying organic matter with carboxyl functions and iron in ambient particles^10,18,39^. These can lead to aerosol anoxia and a consistent source of aerosol-borne CCRs and ROS.

A large radical source due to iron carboxylate photochemistry has important implications for other atmospheric photoactive aerosol components and the lifetime of air mass tracer compounds, pathogens or toxic compounds posing a health risk. Common atmospheric brown carbon compounds such as imidazoles^41,42^, quinone containing compounds^6,43^, or humic-like substances (HULIS)^44^ are photoactive and may limit O_2_ penetration if they produce sufficient radical numbers with low diffusion coefficients. Secondary organic aerosol particles are highly viscous^11,12,25,40^ and photochemical aging may generate radical compounds within them that are preserved. Therefore, we strongly suggest that radical production and persistence along with anoxia may be possible with all photoactive aerosol compounds given sufficient concentration and aerosol viscosity to cause anoxia due to both reaction and diffusion limitations. The transition region for CCRs and ROS at intermediate RH as revealed in Fig. 3 and Supplementary Fig. 9 delineates conditions where radical concentration within particles is still high, but their mobility is substantial enough to exert a significant role in condensed phase aging, e.g., impacting the chemical turnover of harmful organic toxins or tracer compounds for aerosol source apportionment. This transition also coincides with the RH range where the lifetime of viruses suspended in exhaled human respiratory particles is shortest^45^. Exhaled particles of lung-lining fluid are composed of, in part, proteins, saccharides, and iron, which may be highly viscous, susceptible to photochemical radical buildup and impact pathogen viability. Quantifying the link between environmental parameters and aerosol photochemistry as done for the iron carboxylate complexes here will greatly improve our understanding of condensed phase chemistry, radical process, and controls on human exposure to particulate matter that are detrimental to health.

## Methods

### Scanning transmission X-ray microscopy coupled to near-edge X-ray absorption fine structure (STXM/NEXAFS) spectroscopy experiments

We recently developed a photochemical reactor at the PolLux beamline^46^ of the Swiss Light Source to expose particles containing citric acid and Fe^III^Cit to UV light under atmospherically relevant RH and *T*^47,48^. The main advantage of STXM/NEXAFS spectroscopy is the coupling of high-spatial resolution and chemical selectivity for organic carbon functionality and iron oxidation state, and effectively mapping chemical reactions in particles in real-time and in situ^46,49,50^. All particles in the three STXM/NEXAFS experiments were exposed to O_2_ at a partial pressure of 112, 107, and 104 mbar, at RH = 40, 50, and 60%, respectively. Helium was the carrier gas for O_2_ and H_2_O with a total flow of 20 mL min^−1^ at standard temperature and pressure. The total pressure in the environmental chamber was 150 mbar. The iron oxidation state within single particles containing Fe^III^Cit and citric acid was measured using STXM/NEXAFS. A solution having *M*_r_ = 1.0 was prepared and nebulized to generate droplets that were subsequently dried at RH < 30% and impacted onto X-ray transparent membranes^51^. These particles on membranes were then placed in an environmental chamber set inside the X-ray microscope^47^. At all steps, we avoided ambient light and humidity exposure and kept particles in a dry, dark vacuum container prior to use. The time between particle generation and use in STXM/NEXAFS experiments was <2 h. We used STXM/NEXAFS to measure transmitted X-ray photons through a square spot, or pixel, 35 × 35 nm^2^ in size^46^. A 5 × 5 µm^2^ image contained 20,400 pixels, each making up either the transmitted photon count mapped across a particle, *I*, or quantifying the background signal, *I*_0_. From this, the optical density, OD = -ln(*I*/*I*_0_), at each pixel was determined. Acquiring multiple images as a function of X-ray energy yielded a NEXAFS spectrum, which was used to obtain absorption peaks of Fe^III^Cit over the L-edge of iron (700–735 eV) and the K-edge of carbon (280–320 eV). Additionally, we imaged OD at two X-ray energies, 707.8 and 709.5 eV, which correspond to the peak resonant X-ray absorption by iron(II) and iron(III) species, respectively. Energy calibration and determination of non-resonant absorption followed previous work, including a procedure to evaluate X-ray beam damage and avoid it entirely^51^. Image analysis was based on publicly available software tools^50^. Using the parameterization in Moffet et al.^39^ and following an imaging procedure in Alpert et al.^51^, we mapped the iron(III) fraction out of the total iron, *β*, with a time resolution on the order of minutes. Maps were used to derive 2-D *β*-profiles (Fig. 1c, d) following Alpert et al.^51^, by averaging *β* over the perimeter pixels (1 pixel from the perimeter) and those concentric pixels toward the center (>2 pixels from the perimeter). In line with previous studies^51–56^, our particles were determined to be half-spheres, evident from their morphology and OD profiles of particles compared with known spherical polystyrene particles. We note that a single profile, i.e., one color in Fig. 1c, d correspond to a single image of multiple particles (5–20) with >10,000 pixels, where each pixel quantifies iron oxidation state over an area of 1.2 × 10^−3^ µm^2^. Uncertainty in *β* was determined as, Δ*β* = ±0.07 or propagated from the photon counting error, whichever was larger^51^. Prior to illumination, initial *β-*profiles were determined. A UV-LED fiber optic coupling to the STXM chamber was custom-built utilizing UV compatible components and optics, which resulted in a uniformly illuminated sample area with a measured power density of 3.6 ± 0.6 mW mm^−2^ in the narrow wavelength range of 364–370 nm, equivalent to *j* = 2.2 ± 0.04 × 10^−3^ s^−1^ when accounting for the absorption cross section. Illumination for all samples then lasted for 15 min with a UV LED. Immediately after the light was switched off, we continually mapped *β* over multiple particles while re-oxidation took place under a constant RH and O_2_ gas phase concentration, unless otherwise specified. A different procedure is outlined in the Supplementary Information for the data specific to Supplementary Fig. 4 in order to elucidate the radical lifetime. Briefly, this involved photochemical reduction in helium, without the presence of O_2_, and waiting 35 min or 9 h before O_2_ was introduced.

### Electrodynamic balance (EDB) experiments

Experiments using an EDB^28,57^ were performed to measure the mass loss of aqueous particles irradiated by a frequency-doubled diode laser with a wavelength of 473 nm in the visible. In the experiments, the power density was 40 mW mm^−2^ at all RH except for RH = 47%, where it was 20 mW mm^−2^ (*j* = 4.3 × 10^−3^ and 2.2 × 10^−3^ s^−1^, respectively). Aqueous solutions having *M*_r_ = 0.05 were prepared and used to generate an aqueous particle injected into the *RH* and *T* controlled EDB chamber. The injected particle was given ample time to equilibrate with water vapor in an O_2_ atmosphere at a pressure of 800 mbar. The aqueous solution and particle were kept in the dark at all steps prior to irradiation. Before and after the laser was switched on, the particle radius change was monitored over time and the mass loss determined. For each investigated *RH*, a new particle from a freshly prepared solution was used. The EDB applies a high AC voltage across two electrode rings and a DC voltage across hyperbolic end-caps to generate an electric field to levitate particles. The electric field was adjusted automatically to compensate the gravitational force on the particle as its size changed. The refractive index and size of spherical particles were determined from measurements of backscattered light from a broad-band LED centered around 640 nm. The radius loss in Fig. 2a was determined from the ratio between the particle radius over time, *r*(*t*), and the radius prior to illumination, *r*_0_ = *r* (*t* = 0). Due to the high accuracy of determining mass and radius loss, the main source of error was due to the unknown change in refractive index from any changes in composition during irradiation.

### Coated wall flow tube (CWFT) experiments

The release of HO_2_ into the gas phase was measured in CWFT experiments through the efficient scavenging reaction with excess NO^42,58,59^. Films were generated by dispensing a known volume of aqueous Fe^III^Cit and citric acid solutions with *M*_r_ from 0.01 to 0.11 in Duran glass tubes 1.2 cm in diameter and 50 cm long. The error on *M*_r_, Δ*M*, was ±7% on its measured value. Solutions and prepared films were always kept in the dark prior to use. Calculated film thickness was between 0.2–1.8 µm with an approximate ±20% uncertainty and accounted for water uptake at a given RH. When thickness was varied, *M*_r_ = 0.0784 and RH = 38.5%. When *M*_r_ was varied, thickness was 1.1 µm and RH = 37%. Films were allowed to equilibrate to RH prior to the start of an experiment. Uncertainty in RH was ΔRH = ±2%. A constant flow rate of N_2_ and O_2_ was used, typically at a 20% O_2_ content. We also performed O_2_-dependent experiments by varying the partial flow rates, but maintaining a constant total flow, either 1.0 or 0.5 L min^−1^. Prior to illumination, a baseline NO concentration was determined. Illumination using up to 7 fluorescent tubes were then switched on simultaneously causing a drop in NO concentration. This drop was used to calculate the HO_2_ release from the film following a previous procedure^42^. The NO conversion was routinely checked to exclude processes involving NO and HO_2_^42,58^, such as a loss due to O_3_ formation, a production due to the photolysis of NO_2_ and the return of HO_2_ to the film. We note, gas phase loss of HO_2_ with volatile organic compounds is highly unlikely to affect NO conversion as known reaction rates are far too slow. We cycled all films with intervals of UV light switched off and on for approximately 0.5–2 h per interval. Loss of NO and recovery were reproducible to within about 10%, which emphasizes the robustness of our system and our instrument precision. The light output of our fluorescent tubes had a broadband spectrum >300 nm in wavelength and extended into the visible spectrum. Utilizing the measured wavelength-dependent irradiance^42^, absorption cross section^33^, and the quantum yield^28^, we calculated that *j* = 9.1 × 10^−3^ s^−1^, which is higher than that used in STXM or EDB experiments.

### Photochemical reaction and diffusion (PRAD) model

The PRAD model was developed to describe the Fe^III^Cit system constrained with experimental data. It utilizes physical and chemical parameters, such as molecular diffusion coefficients and Henry’s law constants for molecular transport and solubility of the multiple species represented, as well as chemical parameters, such as reaction rates and equilibrium constants. All observables in STXM/NEXAFS, EDB, and CWFT experiments are explicitly modeled and are directly compared. The condensed phase is separated in multiple layers each hosting photochemical reactions, chemical reactions and imposing equilibria conditions first, followed by modeling the flux of molecules between the layers. Details of the PRAD model are given in Dou et al.^28^, and those pertinent to this study are reviewed below. Diffusion coefficients of all species were scaled with the diffusion coefficient of water, *D*_w_, i.e., the diffusion coefficient of a species x is *D*_x_ = *D*_w_*f*_*D*x_, where *f*_*D*x_ is a species-specific constant. We have observed that solution viscosity increased significantly when *M*_r_ > 0.05 (Supplementary Fig. 11), therefore we have decreased *D*_w_ with increasing *M*_r_. For example, *D*_w_ was parameterized to increase by a factor of ~4 from *M*_r_ = 0.05 to 1.0^28^. Known equilibrium coefficients for dissociation and complex formation, photochemical reaction rates, and chemical reaction rate coefficients were determined from previous literature^28^. Unknown equilibrium coefficients and rate coefficients were globally fit to all STXM/NEXAFS, EDB, and CWFT experimental results simultaneously^28^. Henry’s law constants for O_2_, ROS, and other volatile compounds were also adjusted to match observations^28^ and were typically about 1 order of magnitude higher than those reported in dilute aqueous solution. Dou et al.^28^ reported an observed mass loss with UV light exposure using a quantum yield, Φ = 1.0, for wavelengths, *λ* < 400 nm^28^. In order to reproduce our observations in the EDB, we used Φ(473 nm) = 0.002 following our previous work^57^. A parameterization of Φ as a function of *λ* was formulated^28^ and used to predict *j* for CWFT experiments and for *j* due to sunlight reaching the Earth’s surface, which was determined with the online TUV calculator, http://cprm.acom.ucar.edu/Models/TUV/Interactive_TUV/, as a function of solar zenith angle.

## Supplementary information

Supplementary Information

## Data Availability

The data that support the findings of this study are publicly available at 10.5281/zenodo.3817356.

## Code availability

The PRAD model code that support the findings of this study can be found in Dou et al.^28^.
