# Supplementary material for: Photolytic radical persistence due to anoxia in viscous aerosol particles
Source: Nat Commun. 2021 Mar 19;12:1769. doi: 10.1038/s41467-021-21913-x (PMC7979739; doi:10.1038/s41467-021-21913-x)
Supplement: Supplementary file 1 — Supplementary Information [file 41467_2021_21913_MOESM1_ESM.pdf]

Supplementary Information for “Photolytic Radical Persistence due to Anoxia in Viscous Aerosol Particles”

Peter A. Alpert[1,*], Jing Dou[2], Pablo Corral Arroyo[1,†], Frederic Schneider[1], Jacinta Xto[3], Beiping Luo[2], Thomas Peter[2], Thomas Huthwelker[3], Camelia N. Borca[3], Katja D. Henzler[3], Thomas Schaefer[4], Hartmut Herrmann[4], Jörg Raabe[5], Benjamin Watts[5], Ulrich K. Krieger[2] and Markus Ammann[1,*]

[1]Laboratory of Environmental Chemistry, Paul Scherrer Institute, 5232 Villigen, Switzerland

[2]Institute for Atmospheric and Climate Science, ETH Zurich, 8092 Zurich, Switzerland

[3]Laboratory for Synchrotron Radiation and Femtochemistry, Paul Scherrer Institute, 5232 Villigen, Switzerland

[4]Atmospheric Chemistry Department (ACD), Leibniz Institute for Tropospheric Research, 04318 Leipzig, Germany

[5]Laboratory for Synchrotron Radiation-Condensed Matter, Paul Scherrer Institute, 5232 Villigen, Switzerland

[†]now at the Laboratory for Physical Chemistry, ETH Zurich, 8092 Zurich, Switzerland

[*]Correspondence to: Peter A. Alpert (peter.alpert@psi.ch) and Markus Ammann (markus.ammann@psi.ch)

Supplementary Discussion

Photochemical Cycling in Particles Composed of Iron(III) Citrate and Citric Acid

A sketch of the photochemical reaction scheme for a particle composed of iron(III) citrate and citric acid is shown in Fig. 1a of the main text. Citric acid complexed with Fe^{3+}, or iron(III) citrate ($Fe^{III}CA$) was used as a model atmospheric photochemical system due to its absorption of UV light in the same range of wavelengths as those hitting the surface of the Earth. In addition, citric acid is a well-established proxy for oxygenated aerosol and known for its increasing viscosity as a function of decreasing *RH* and *T* leading to a reduction in diffusion of reactive and non-reactive trace gases[2,3]. The green to grey color gradient highlights O_2 present near the surface of the particle and anoxic conditions in the bulk. Colored text highlights specific features of the reaction cycle that were experimentally investigated. Purple, blue and red text indicates sensitivity to the measured iron oxidation state from scanning transmission X-ray microscopy coupled to near edge X-ray absorption fine structure (STXM/NEXAFS) spectroscopy experiments, mass loss with electrodynamic balance (EDB) experiments and HO_2 radical production in coated-wall flow tube (CWFT) experiments, respectively. Light absorption is indicated by the yellow wavy lines, followed by ligand-to-metal charge transfer (LMCT), and then dissociation yielding Fe^{2+}, CO_2, and the carbon-centered radical (CCR) in yellow text, $HO\text{-}^{\bullet}CR_2$, where R is CH_2COOH. Reactions are indicated by solid black arrows. Evaporation and uptake, e.g. of CO_2 and O_2, is indicated by dash-dotted arrows. O_2 reaction with CCRs yields the reactive oxygen species (ROS), HO_2 and the ketone compound with 5 carbon atoms, $O{=}CR_2$, of which half is considered to photochemically decay into smaller molecules with 2-4 carbon atoms that are capable of evaporating from the particle. The HO_2 produced in the condensed phase evaporates or reacts with Fe^{2+} to produce Fe^{3+} and a cascade of the other ROS species H_2O_2 and OH. Equilibria between Fe^{3+}, Fe^{2+} and dissociated citric acid are indicated by the dashed arrows. Equilibrium conditions for aqueous citric acid dissociation, non-photoactive iron(III) complexes, the iron(II) complex $Fe^{II}O{=}C(CH_2COO)_2$, the superoxide radical and finally charge balance is not depicted. Other photochemical reactions and the reactions involving O_2^- are not indicated in Fig. 1a of the main text. However, all are

included in the photochemical reaction and diffusion (PRAD) model to describe our experiments and predict CCR and ROS formation. Details on all reactions, equilibrium conditions and species charge balance considered here can be found in Dou et al. (2021)[4].

Iron Oxidation State and X-ray Spectromicroscopy

Here, we describe the physical and chemical parameters pertinent for experiments using STXM/NEXAFS. Citric acid, iron(III) citrate and iron(II) citrate are denoted as CA, $Fe^{III}Cit$ and $Fe^{II}HCit$, respectively, in Fig. 1a of the main text. Reduction of iron(III) to iron(II) by light through LMCT, and reoxidation by O_2 and ROS changed the iron oxidation state within particles. The fraction of iron(III) out of the total iron, β, was quantified by STXM/NEXAFS spectroscopy. These observations were especially valuable because iron reduction and subsequent oxidation by O_2 and ROS exactly reveals the location within particles where

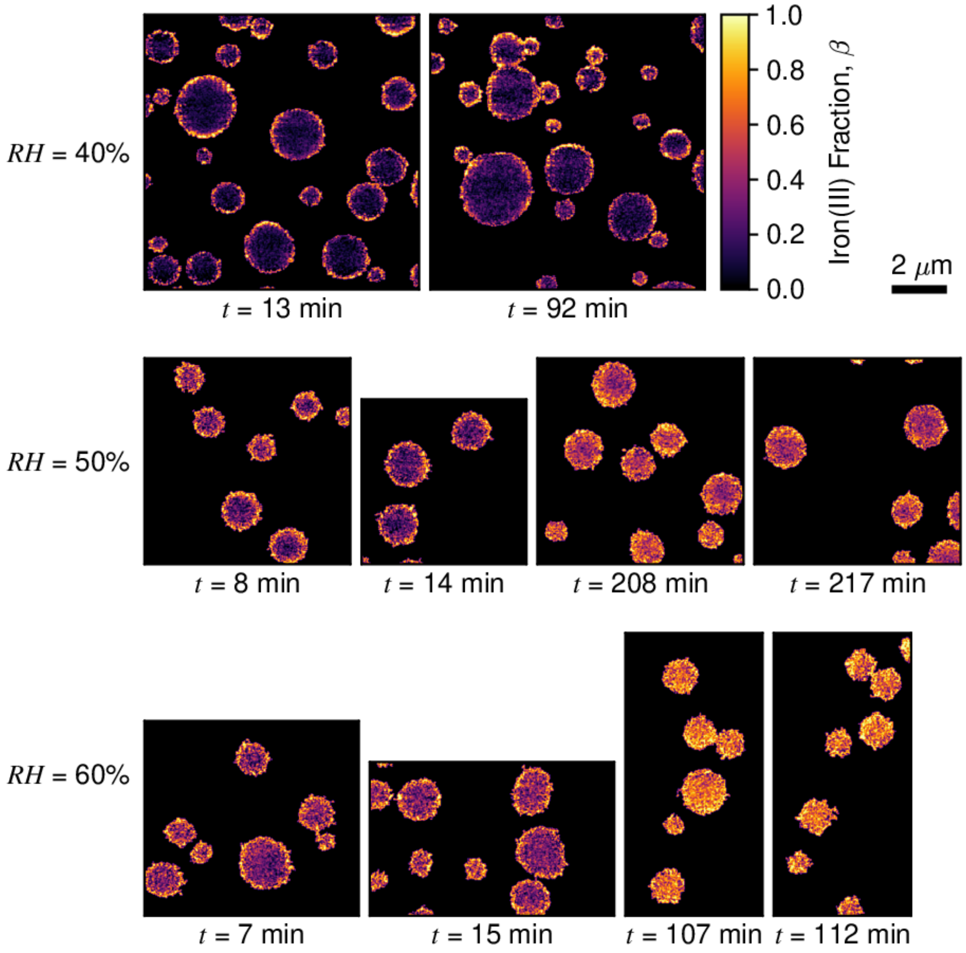

Supplementary Figure 1: Images taken with scanning transmission X-ray microscopy coupled to near-edge X-ray absorption fine structure spectroscopy (STXM/NEXAFS). X-ray images of the iron(III) fraction, β (color scale), acquired from experiments performed at a relative humidity, *RH* = 40, 50 and 60% in the top, middle and bottom rows, respectively. Pixel resolution was 35 × 35 nm^2 and used to calculate 2-D profiles seen in Fig. 1c-e in the main text. The images are smoothed by increasing the pixel resolution and interpolating, and the background was removed (appears as black) for image clarity. At every *RH*, UV irradiation lasted 15 min and then UV light was shut off. The time after UV light was shut off, *t*, is indicated for each image. The scale bar applies to all images.

CCRs and ROS were produced, respectively. Particles underwent 15 min of UV light exposure, then the UV light was switched off, and images of β within particles were acquired over time, *t*, during reoxidation in the dark. Supplementary Figure 1 shows example images at the start and end of reoxidation, where the time after UV was switched off is indicated above each image. These images are 2-D column integrated averages of β taken with 35 × 35 nm^2 spatial resolution, which is tens of thousands of pixels per image. This sheer amount of data yields high statistical significance of β as detailed in our previous study[10]. This information also constrains reaction rates, diffusion coefficients and Henry's law constants of these complexes and their reaction partners[10]. The PRAD model predicts the radial concentration of all iron containing species in spherical particles of a given size. We used this radial information to generate 2-D model STXM images to directly compare with observations, i.e., solid lines and symbols, respectively, in Fig. 1c-e in the main text. Further information about the procedure for calculating β can be found in previous literature[4,6,10].

Measured Mass Loss From CO_2 and Volatile Organic Compounds

After the initial Fe^{III}Cit photochemical reaction, decarboxylation occurred releasing CO_2 to the gas phase and producing a CCR that remained in the particle phase. The mass loss of particles measured by EDB experiments shown in Fig. 2a of the main text was mainly due to the evaporation of CO_2. These data mainly constrained chemical kinetic reaction rates and equilibrium coefficients that produce CO_2 and cycle iron(II) and iron(III). Other parameters, such as diffusion coefficients and Henry's law constants, were also constrained by EDB together with STXM/NEXAFS and CWFT results. The reaction between O_2 and the first generation of CCRs yields HO_2 and $O{=}CR_2$, as previously discussed. This species was assumed to be photoactive, but with a photochemical reaction rate, *j* with units of s^{-1}, 1 - 3 orders of magnitude slower than that of Fe^{III}Cit. Photochemical reactions with C_5 compounds formed more volatile compounds with 2 - 4 carbon atoms. These compounds, C_2, C_3, and C_4 are indicated in Fig. 1a of the main text as evaporating from the particle, which also contributed to the observed mass loss.

Radical Production and Release

Production of HO_2 in a particle or film was due to the reaction between O_2 and CCRs and the reaction between O_2 and iron(II) citrate. HO_2 evaporated from the particle and was observed in the gas phase in CWFT experiments as HO_2 production[12-14]. HO_2 can react with itself with a rate coefficient, k_{SR}, or react with Fe^{2+} to form Fe^{3+}. The production, reaction and evaporation of HO_2 depicted in Fig. 1a of the main text is explicitly treated in the PRAD model and compared with observations. Corral Arroyo et al. (2018)[13] measured HO_2 production in organic films containing photosensitizer compounds and developed a box model to reproduce their results. The authors found that k_{SR} (or reaction R5 in Dou et al. (2021)[4]) was not well-constrained in their model and were required to reduce k_{SR} at low *RH* when compared with k_{SR} = 8.3 × 10^5 M^{-1} s^{-1} in dilute aqueous solution. Here, M is molarity in units of mole L^{-1}. A parameterization of k_{SR} as a function of film water activity, a_w, was proposed, which would effectively increase the modelled HO_2 concentration in films and thus in the air above it. To better understand our results of HO_2 production, we have also investigated HO_2 reactions, and how different parameterizations impact the HO_2 turnover rate detailed in a later section (Supplementary Figure 6). Observations of HO_2 production attained a steady state and are reported as constant values. This implies the persistence of radical production and cycling in particles as a constraint to ROS production and to Fe^{III}Cit photochemistry.

Reoxidation in STXM/NEXAFS Experiments

It is important to note that β derived from the PRAD model shown in Fig. 1c-e of the main manuscript was determined from radial profiles shown in Supplementary Figure 2a-c. This was performed following the procedure of Alpert et al. (2019)[10] by translating radial profiles to 2-D column-integrated profiles (images) with the same pixel resolution as STXM/NEXAFS experiments. At *RH* = 40% (Supplementary Figure 2a, d and g) for *t* = 0 - 15 min when UV irradiation was switched on, iron was reduced, CCRs were produced and oxygen was immediately depleted. UV light was switched off from *t* = 15 - 108 min and reoxidation in the dark proceeded. However, this was slow, as indicated by the very little change in β for *t* > 15 min and due to O_2 being limited to the near-surface when UV light was both on and off. O_2 penetrated about 2.5 nm from the particle surface until its concentration was an order of magnitude less than its maximum value, $[O_2]_{max} = 4.2 \times 10^{-3}$ M. After UV light was switched off, O_2 penetration increased to 13 nm. Therefore, most of the particles remained anoxic, and it was this lack of O_2 that led to a very low turnover of CCRs. Likewise,

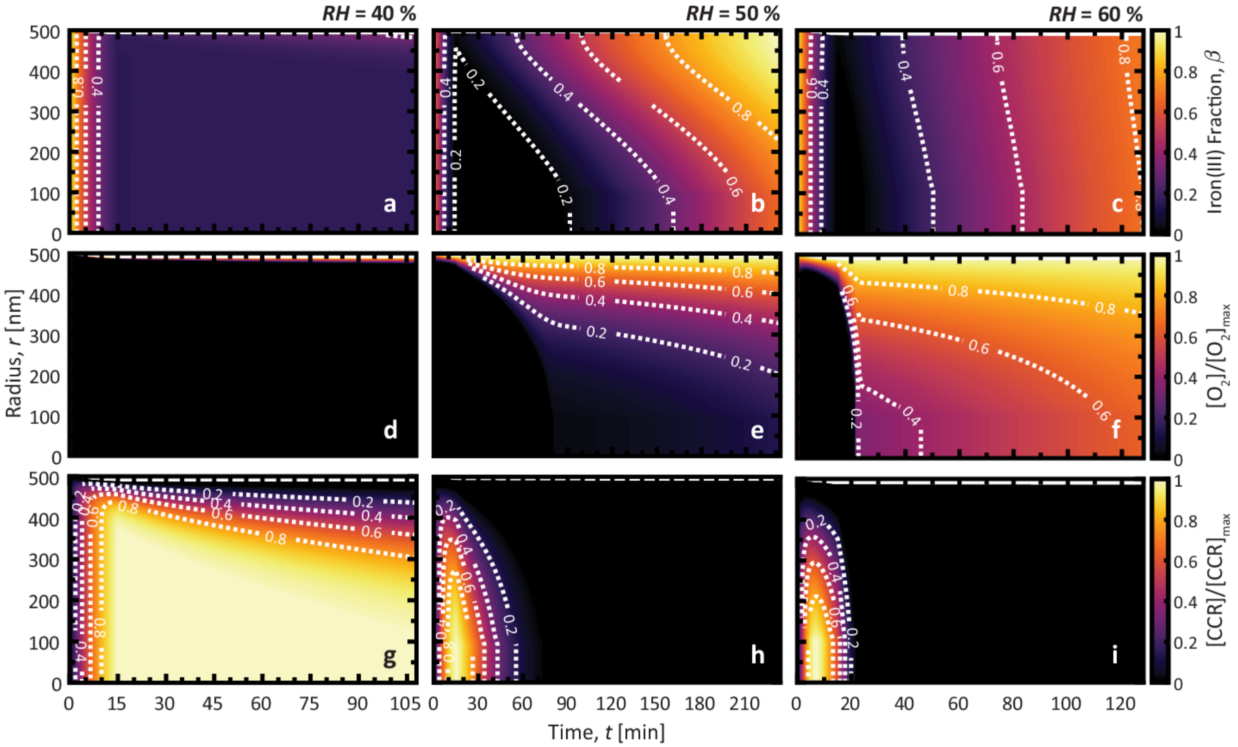

Supplementary Figure 2: Profiles of the iron(III) fraction, β (a-c), normalized O_2 concentration, $[O_2]/[O_2]_{max}$ (d-f) and the normalized carbon centered radical (CCR) concentration, $[CCR]/[CCR]_{max}$ (g-i) for the photochemical reaction and diffusion (PRAD) model. These profiles are exactly the lines in Fig. 1 of the main text employing a semi-spherical geometry. Three model runs were performed at a relative humidity, *RH* = 40% **(a, d** and **g)**, 50% **(b, e** and **h)** and 60% **(c, f** and **i)**. For all *RH*, the initial particle radius at time, *t* = 0 min was 500 nm and the mole ratio between iron(III)-citrate and citric acid was M_r = 1.0. During *t* = 0 - 15 min, UV irradiation was on. At *t* = 15 min, UV irradiation was switched off. Contours as dotted lines are shown expect for panel **d** for clarity. Note that the *t* axis is different for the different *RH* values. At *RH* = 40%, the maximum time is t_{max} = 108 min, the maximum CCR concentration is $[CCR]_{max}$ = 2.3 mole L^{-1} and the maximum O_2 concentration is $[O_2]_{max} = 4.2 \times 10^{-3}$ mole L^{-1}. At *RH* = 50%, t_{max} = 223 min, $[CCR]_{max}$ = 1.4 mole L^{-1} and $[O_2]_{max} = 4.1 \times 10^{-3}$ mole L^{-1}. At *RH* = 60%, t_{max} = 128 min, $[CCR]_{max}$ = 0.9 mole L^{-1} and $[O_2]_{max}$ = 4.0×10^{-3} mole L^{-1}.

ROS formation was very low since there was very little O_2 in the particles. The profiles of CCRs at *RH* = 40% in Supplementary Figure 2g show that they persisted at high levels, again due to the fact their diffusion coefficient was low enough to prevent them from moving to the O_2 rich surface.

The surface of the investigated particles never reoxidized completely, i.e., values of β never reached close to 1.0 in the topmost model layer. This is due to the fact that slow reoxidation occurred, in part due to the O_2 concentration being far less than CCRs or iron(II) citrate complexes. A very sharp gradient would require either Henry's law coefficient for O_2, H_{O_2}, or the reaction rate coefficient with CCRs and iron(II) citrate complexes to be much greater than what was used in the model. Yet, this would result in a very poor representation of our observations in general, and parameters chosen in Dou et al. (2021)[4] were more suitable overall. Oxygen absence in the bulk of a particle meant that only surface layers could have hosted transitions from iron(II) to iron(III) followed by iron complexation with other carboxyl functional groups and thus becoming light absorbing molecules again. This includes not only Fe^{III}Cit, but potentially other metal-organic complexes with organic products, i.e., the C_3 - C_5 species. This exactly means that photochemical cycling should depend on particle size, depth within particles and how molecules diffuse radially. In other words, how much iron(II) is oxidized back to iron(III) integrated over the bulk of the particle will depend on particle size, while the local (spatially resolved) turnover will depend on the radial position.

At *RH* = 50%, modelled profiles indicated a faster chemical change over *t* with concentration gradients extending further into the particle interior than at *RH* = 40%. Supplementary Figure 2b shows that β values changed by 0.4 over 500 nm, i.e., from the surface to center of the particle. During UV irradiation, the particle was anoxic with O_2 reaching 16 nm from the particle surface. This is a similar situation as for *RH* = 40%, and implies that continual production and reaction of CCRs and iron(II) citrate kept O_2 near the surface of particles. When UV was switched off, however, O_2 began to move through the particle and reacted with both CCRs and iron(II) citrate. However, the reaction with CCRs was the dominant sink for O_2, which led to depletion of CCRs throughout the whole particle after 80 min. This is in contrast to experiments at *RH* = 40%, in which radical concentration dropped to about 65% of its highest value over the same dark reoxidation time. Higher *RH* resulted in a greater water concentration, which acted as a plasticizer in the particles. This resulted in decreased viscosity and increased diffusion coefficients of all reactants. Therefore, chemical reaction that occurred within the particle bulk at higher *RH* would be less limited than at lower *RH*, in line with our observations. At *t* > 80 min, a gradient in O_2 was still present in the PRAD model and maintained only by the slower reaction with iron(II) citrate complexes.

At *RH* = 60%, β was observed to be fairly uniform over *t* as shown in Fig. 1e of the main text. Radial profiles of β in Supplementary Figure 2c were close to homogeneous, although O_2 was still limited to near-surface layers shown in Supplementary Figure 2f. In fact, in all EDB and CWFT experiments at our lowest investigated M_r = 0.01, anoxic conditions and radical persistence was predicted by the PRAD model during light exposure. However, the lifetime of CCRs depended on *RH* and *T*, which is discussed in detail in a later section. After UV light was switched off, O_2 quickly moved through the whole particle where it reacted with CCRs and iron(II) complexes. After CCRs were depleted, a small gradient in O_2 remained. CCRs

produced photochemically were reacted away more quickly than at lower *RH* and existed only when and where O_2 was not present. These results support our hypothesis that organic radicals persisted and were unreacted in highly viscous particles with lifetimes on the order of hours, but not in particles with lower viscosity characterized with higher diffusion coefficients. The cause of the propagation of oxidation through reaction of CCRs to peroxy radicals and their depletion is diffusion and reaction limitations. This points toward anoxic conditions in CCR-rich particles where diffusion coefficients are low, and the inability of CCRs and ROS generated in the interior of particles to escape.

Uncertainty and Model Sensitivity for STXM/NEXAFS Experiments

Model Sensitivity on Adjusted Parameters

Sensitivity and uncertainty analysis of the PRAD model and STXM/NEXAFS experiments are given in Supplementary Figure 3. In the PRAD model, diffusion and reaction of iron containing species, i.e., iron complexes and free iron, and O_2 and CCR molecular transport play major roles in establishing our observed time evolving gradients. The uncertainty in β values from STXM/NEXAFS experiments is due to X-ray photon counting statistics, X-ray image alignment, and particle-to-particle variability[10]. Evaluating the variability in β due to environmental parameters and initial conditions would require multiple experimental trials using multiple samples at the same conditions. At an X-ray beamline, this is not possible due to strict time constraints, and therefore, we have utilized the PRAD model to investigate the sensitivity in β to our experimental uncertainty in *RH*, the measured initial iron(III) fraction, β_0, and irradiance. In other words, PRAD model input parameters were varied according to their experimental uncertainty, and the model response in β was determined. For increasing *RH* between 40 - 60%, reoxidation occurred much faster and profiles in β became more uniform (Fig. 1 in the main text). The error in *RH* was $\Delta RH = \pm 2\%$, the error in β_0 was $\Delta\beta_0 \pm 0.07$ and the measured power density was 3.61 ± 0.60 mW mm^{-2}, which is equivalent to $j = 2.16 \pm 0.35 \times 10^{-3}$ s^{-1}.

Supplementary Figure 3 shows the PRAD model results when *RH* was decreased or increased by 2%, β_0 was decreased or increased by 0.07 and j was increased or decreased by 0.35×10^{-3} s^{-1}, respectively. The results show that the model is fairly sensitive to these input parameters as seen in the lightly shaded regions in Supplementary Figure 3. The width of the grey shaded box is about the same as all other colors indicating that the largest contributor to the total error may be due to $\Delta\beta_0$. At $RH = 50\%$, the shading becomes wider than $\Delta\beta_0$, likely due to *RH* altering the reoxidation rate over t. We point out that minutes after light was shut off at $RH = 50$ and 60%, the PRAD model predicted β at lower values than observed. This could mean that j during UV exposure may have been slightly too high. We have used a quantum yield, $\Phi = 1.0$, for iron(III) reduction and decarboxylation determined from Dou et al. (2021)[4]. STXM/NEXAFS measurement may be impacted by a reverse reaction in which the pre-dissociation complex undergoes internal conversion back to its ground state[15]. However, this was a minor effect as described in Dou et al. (2021)[4]. Overall, calculating j using absorption coefficients from literature[15] was sufficient to reproduce results in Dou et al. (2021)[4] and our results presented here within our experimental uncertainty and model sensitivity.

Limitations on Reactions With O_2

Linear changes in *RH* result in exponential changes in diffusion coefficients and for this reason, observable gradients in β were highly sensitive to *RH* in a narrow range between 40 - 60%. This may coincide with a transition between different limiting cases in the photochemical reaction cycle[16,17]. In addition, different limiting cases may exist between light-on and light-off time intervals. It is well known that multi-phase reactions involving ROS species, such as O_3 or OH radicals, can follow a reacto-diffusive kinetic regime, i.e., ROS taken up by a particle from the gas phase remains confined to a very thin layer beneath its surface compared to its radius[18-20] provided that they react reasonably fast with the organic components. Following this general limiting case, $l_{rd} = (D/k^I)^{1/2}$ is the reacto-diffusive length of a specific reactant[4] and indicates a $1/e$ reduction in its concentration, where D is its diffusion coefficient and k^I is the first order loss rate with units of s^{-1}. We note that a reacto-diffusive limitation has been moderately successful to approximate O_3 reaction in our previous studies with a condensed phase organic reactant[18] and for trace metal reoxidation in an organic matrix[10]. To assess whether or not l_{rd} can accurately represent O_2 penetration depths in our photochemical system, we have compared the gradients derived from the PRAD model (Supplementary Figure 2) of the reacting species, O_2, CCRs and iron(II) citrate with their calculated l_{rd}.

Supplementary Table 1 gives the values of l_{rd} for O_2 when UV light was on and off reacting either with CCRs or iron(II) citrate (listed as Fe^{II}HCit). As previously mentioned, the reaction O_2 + CCR yields HO_2 and the ketone compound, $O{=}CR_2$, where R stands for CH_2COOH (see Fig. 1a in the main text). As a comparison, we have also calculated the length, l_e, over which O_2 was reduced by a factor of $1/e$ using the O_2 profiles from the PRAD model in Supplementary Figure 2. Irrespective of light on or off at *RH* = 40%, l_{rd}

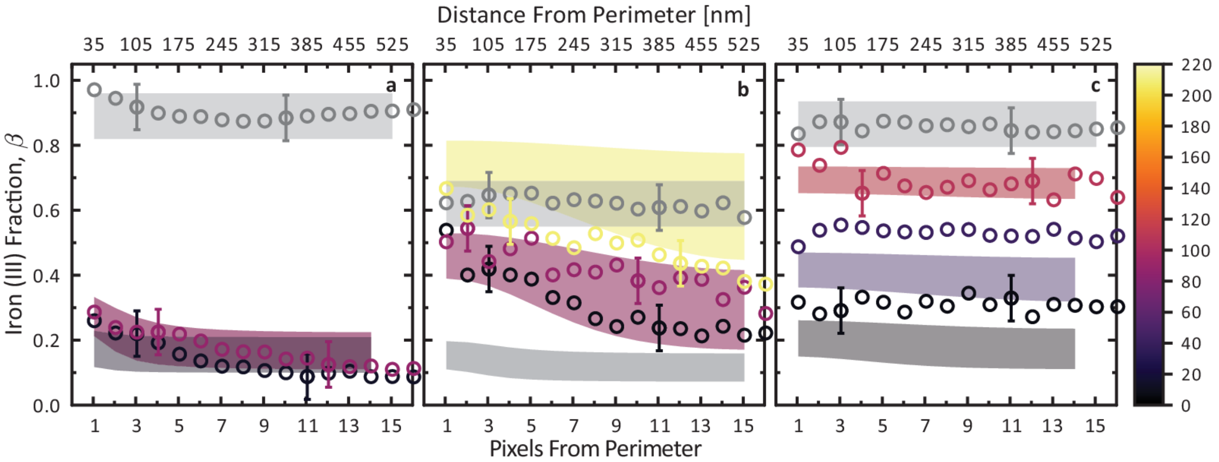

Supplementary Figure 3: Model sensitivity on iron(III) fraction, β, experimental error. Measured β over time, *t* (color scale), as a function of the pixels and distance from the particle perimeter is reproduced from Fig. 1 of the main text for subset of data at a relative humidity, *RH* = 40, 50 and 60%, shown in **a**, **b** and **c**, respectively. The grey symbols are measured prior to light exposure and are the initial iron(III) fraction, β_0. For each experiment, model input parameters were adjusted by their experimental uncertainty. An upper sensitivity limit was obtained by increasing *RH* by 2%, increasing β_0 by 0.07 and decreasing the photochemical reaction rate, *j*, by 16%. A lower sensitivity limit was obtained by decreasing *RH*, decreasing β_0 and increasing *j* by the same amount. The resulting range of modelled β is shown as light shadings with their colors corresponding the same color as the symbols.

was < 1 nm when reacting with CCRs and on the order of 10^2 for the reaction with $Fe^{II}HCit$. l_e from the PRAD model was on the order of 10^0-10^1 nm between the time when light was on and off, respectively. This implies that l_{rd} may not accurately estimate the actual O_2 penetration into the particle bulk. Additionally, l_{rd} for CCRs and $Fe^{II}HCit$ was < 1 nm and > 200 nm, respectively, and again shows that their reaction with O_2 both contribute to the degree of O_2 penetration. For multiple species reacting and diffusing, the reacto-diffusive length concept is not generally valid as shown here. Despite this, l_{rd} for the reaction with iron(II) citrate was closer to the order of magnitude of l_e and much smaller than the particle radius, r, which supports anoxic conditions in the bulk. At RH = 50% and 60% when UV light was on, the particle remained anoxic with l_e = 11 nm and 28 nm, respectively, both of which are much smaller than r. However, l_{rd} determined from the reaction with CCRs or $Fe^{II}HCit$ under and over predicted l_e, respectively. When light was switched off after a long time, all CCRs were depleted and O_2 reactions only with iron(II) citrate occurred. Generally, O_2 was used up by photochemically-generated CCRs over very short lengths, but iron(II) citrate also limited the reaction over length scales of tens to hundreds of nanometers.

Supplementary Table 1: Evaluation of reacto-diffusive parameters. Diffusion coefficients of O_2, D_{O_2}, and rate coefficients for reaction with carbon centered radicals (CCR), k_{O_2+CCR}, and iron(II) citrate, $k_{O_2+Fe^{II}HCit}$, are indicated. Concentrations averaged over a particle with a radius initially, r_{max} = 500 nm, are $[CCR]_{avg}$ and $[Fe^{II}HCit]_{avg}$. Calculated reacto-diffusive lengths of O_2 from the reaction with CCRs and iron(II)-citrate, $Fe^{II}HCit$, are $l_{rd,CCR}$ and $l_{rd,Fe^{II}Cit}$, respectively. The distance at which O_2 concentration drops by a factor of $1/e$ determined from our model is l_e. Time, t, refers to the time after UV light was switched off, and M is molarity in units of mole L^{-1}.

	$RH = 40\%$		$RH = 50\%$		$RH = 60\%$	
D_{O_2} / nm^2 s^{-1}	59		8.2×10^2		3.9×10^3	
k_{O_2+CCR} / M^{-1} s^{-1}			1.0×10^6			
$k_{O_2+Fe^{II}HCit}$ / M^{-1} s^{-1}			5.0×10^{-2}			
	$t < 15$ min	$t > 90$ min	$t < 15$ min	$t > 90$ min	$t < 15$ min	$t > 90$ min
$[CCR]_{avg}$ / M	1.5	1.1	0.50	–	0.25	–
$[Fe^{II}HCit]_{avg}$ / M	2.1	2.7	2.2	1.4	1.8	1.1
$l_{rd,CCR}$ / nm	6.5×10^{-3}	7.2×10^{-3}	4.0×10^{-2}	$> r_{max}$	1.3	$> r_{max}$
$l_{rd,Fe^{II}Cit}$ / nm	24	21	88	1.1×10^2	2.2×10^2	2.7×10^2
l_e / nm	1.8	8.8	11	1.4×10^2	28	$> r_{max}$

Elucidating Radical Lifetime

In addition to CCRs reacting with O_2 to form C_5 compounds and HO_2, it is possible that CCRs react with themselves leading to stable compounds thus terminating radical reaction chains. Depleting CCRs this way may result in slowing photochemical cycling. In particular, CCRs may recombine to form higher molecular weight oligomers when O_2 is depleted[21,22]. In general, oligomers have been shown to form and be present in air[23]. Additionally, unimolecular radical reactions are known to eliminate substitutions, such as CO_2 and HO_2, which occurs in our system. We speculate that if CCRs undergo elimination reactions, they would contribute to the production of e.g., CO_2, small radical compounds and volatile organic compounds.

We have performed additional sets of STXM/NEXAFS experiments to support our conclusion for long radical lifetime, and that radical recombination is likely to play a minor role in viscous particles. Supplementary Figure 4 shows data taken from the bulk reoxidation experiments from Dou et al.[4] used to constrain parameters for the PRAD model shown as green symbols. This bulk averaged value of β is taken from the same experiment as shown in Fig. 1e of the main text. We fitted a 1st order reoxidation rate, k_{reox}, at RH = 60% and obtained 1.3×10^{-4} s^{-1}. In an additional experiment (red symbols), a fresh sample was irradiated with UV light in humidified helium at RH = 60% to reduce the iron, followed by the particles resting 35 min after the UV light was switched off without O_2 present. Afterward, O_2 was introduced, and values of β averaged over all particles was observed to increase at a slower rate, $k_{reox} = 4.2 \times 10^{-5}$ s^{-1}. Another additional experiment was performed similarly, except the wait-time in humidified helium was 9 hr and at RH = 40%. After O_2 was added, β was measured for about 75 min (open black circles), then the sample RH was increased to 60% until ~270 min (filled black symbols). The reoxidation rate was much slower at $k_{reox} = 8.5 \times 10^{-6}$ s^{-1}. This supports the notion that radical recombination reactions may occur, as mentioned in the main text, and possibly significant after a long time at RH = 60%. At lower RH, radical recombination rates may have been much slower. This also implies a long radical lifetime, in support of our conclusion that CCRs are quickly formed and persist for a long time in particles depleted by O_2.

Although the evidence in Supplementary Figure 4 supports long CCR lifetimes, it cannot quantify radical lifetime due to the fact that CCRs are not directly measured. In addition, there are other processes

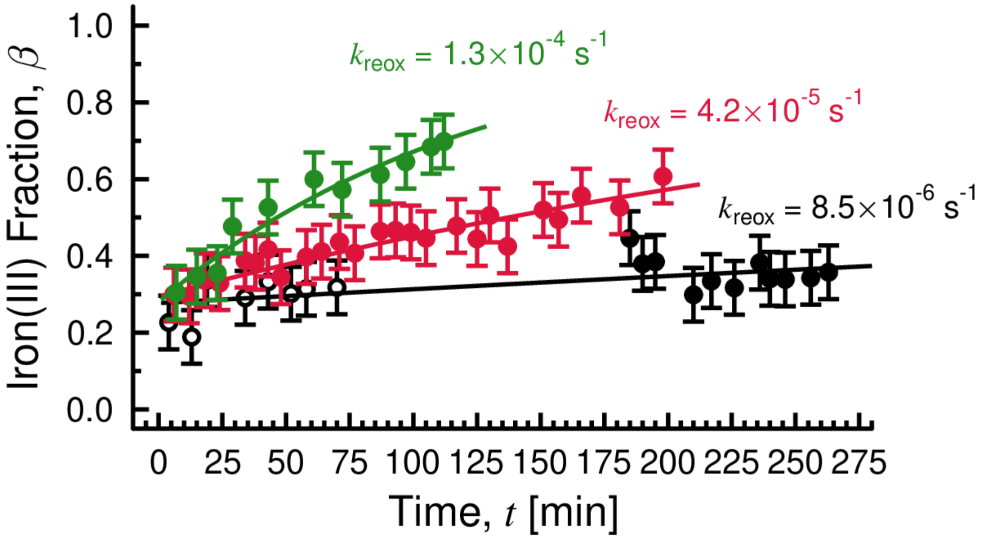

Supplementary Figure 4: Increase in the particle iron(III) fraction, β, during O_2 exposure. Three different samples are shown in different colors, where the fitted exponential reoxidation rate, k_{reox}, is given. Black symbols show a 4 step experiment in which particles were first photochemically reduced in helium (without the presence of O_2) at a relative humidity, RH = 40% (before time, t = 0 min). Second, the particles remained in humidified He (RH = 40%) for 9 hr (still before t = 0). Third, the particles were exposed to O_2 (open circles). Fourth, the RH was changed to 60% (filled circles). In a similar experiment, particles in humidified helium (RH = 60%) were irradiated with UV, remained for 35 min, then O_2 was introduced to start reoxidation shown as red symbols. The start time, t = 0 min, refers to the time when particles were first exposed to O_2. The green symbols are taken from Dou et al.[4] during which irradiation and reoxidation was performed in humidified He and O_2. For the green symbols, the time when UV irradiation was switched off is t = 0 min.

that may still need to be considered preventing an accurate determination. These processes include radical recombination reactions as discussed above that are typically fast, e.g., the decay rate of radicals formed from OH reacting with citric acid is on the order of 10^8 and 10^6 M s^{-1} at a pH of 1 and 7, respectively[24]. In highly viscous particles, however, an upper limit of reaction rates may only be as large as the bulk diffusion controlled rate[25]. Diffusion coefficients of CCRs were estimated to be quite low, on the order of 10^{-18} cm^2 s^{-1} at *RH* = 40%. This results in a diffusion-controlled self-reaction rate (an upper limit) calculated to be on the order of 10^2 M^{-1} s^{-1}. Furthermore, the lifetime of some radicals can be on the order of days in the presence of O_2 when stabilized with transition metals, known as environmentally persistent free radicals (EPFRs)[5,26-28]. If this occurred in our experiments, the lack of reoxidation with O_2 in Supplementary Figure 4 may be the result of radical stabilization with iron similar to EPFRs[5,26-28]. Reaction of iron(II) complexes with peroxy radicals is also fast (on the order of 10^6 M^{-1} s^{-1}), and can form non-radical iron(III) complexes[29]. Although, the diffusion coefficients of these species should be quite low and may be limited by a bulk diffusion controlled rate. New alkoxy radicals can be formed though iron oxidation with peroxides[30]. Reactions involving compounds in the peroxide family require O_2 and would be in competition with ongoing photochemical cycling in near-surface layers of particles. Alternatively, an electron transfer reaction from radicals to iron(III)-glyoxalic acid complexes in dilute aqueous solution that does not require O_2 has been proposed to reduce iron and thus deplete radicals[31]. However, we have observed little change in β (black symbols in Supplementary Figure 4) when photochemically reduced particles sat for 9 hours without O_2. Finally, it cannot be ruled out that a favorable chemical process at low *RH* (lower water content) could have sequestered radicals into more stable compounds independent of viscosity that may have influenced diffusion coefficients when changing humidity from 40% to 60% (black symbols). For these reasons, we cannot claim a radical lifetime explicitly or even that iron-stabilized radicals persisted for >9 hr in our experiments. We find that much more work is need to resolve these issues and argue from the available evidence that CCR lifetime is long, allowing them persist at high concentration.

HO_2 Production in Films

HO_2 Production as a Function of Oxygen Content in Air and Light Intensity

The production of HO_2, $P_{\mathrm{HO_2}}$, as a function of the mole ratio between Fe^{III}Cit and citric acid, M_r, and the film thickness in CWFT experiments was presented in Fig. 2b of the main text. At steady state, $P_{\mathrm{HO_2}}$ ranged from 0.6 - 5.5 × 10^{11} molec. cm^{-2} min^{-1}. Even when iron concentration was at the lowest we investigated, i.e. M_r = 0.01, the PRAD model predicted anoxic conditions and high radical concentrations as shown in Supplementary Figure 5. Additionally, the HO_2 profile shown in Supplementary Figure 5c reveals that its concentration is at its highest at the intersection of the regions with high O_2 and CCR concentrations. This is due to the largest HO_2 source being due to the reaction of O_2 and CCR, which will be discussed in a later section (see Supplementary Figure 7).

We have additionally measured HO_2 production as a function of oxygen partial pressure and light intensity shown in Supplementary Figure 6, which also includes an analysis on the model derived HO_2 turnover rate. Flows of N_2 and O_2 were varied at ratios of 1.0, 0.8, 0.6, 0.4, 0.2 and 0.0, which were used to calculate the oxygen partial pressure out of the total pressure. The O_2 partial pressure was an input parameter for the PRAD model. The CWFT utilized 7 fluorescent tubes, of which 1, 3, 5 and 7 were used

for the light intensity dependent experiments corresponding to j = 0.9, 2.7, 4.6 and 6.4 × 10^{-2} s^{-1}, respectively. Supplementary Figure 6a shows that P_{HO_2} decreased only slightly as j decreased (green symbols). This is likely due to the main driver of P_{HO_2} being photochemically-generated CCRs reacting with O_2. The fact that P_{HO_2} is fairly insensitive to j is due to the fact that Fe^{III}Cit was rapidly depleted so that overall productivity of HO_2 is limited by the O_2 supply, which remained constant. We note that P_{HO_2} was never measurable in the dark. P_{HO_2} was fairly independent of the O_2 partial pressure achieving a value of about 1.2 × 10^{11} molec. m^{-2} min^{-1}, varying less than 7% and showing no particular trend (black symbols in Supplementary Figure 6a). The production of ROS (and thus also HO_2) is entirely dependent on O_2 content in the particle, which certainly increased by a factor of 5 between the investigated O_2 partial pressures of 145 - 730 torr. The PRAD model equilibrates the first near-surface layer of films and particles with Henry's law, and thus the O_2 concentration was a maximum there with $[O_2]_{max}$ = 3.4 × 10^{-2} and 6.7 × 10^{-3} M for the highest and lowest O_2 content, respectively. P_{HO_2} was below detection limit when O_2 was not present, which confirms that there does exist some O_2 dependence, but not in our investigated range.

HO_2 Turnover

To better understand our results, we have investigated the sensitivity of P_{HO_2} predicted by the PRAD model to the choice of the HO_2 self-reaction rate constant, k_{SR}. The solid line in Supplementary Figure 6a is from the PRAD model where k_{SR} was adjusted to match observations, while the shading is the sensitivity to a factor of 5 in k_{SR}. P_{HO_2} was also determined using a parameterization of k_{SR} as a function of RH from Dou et al. (2021)[4] shown as the dashed line and using a constant value k_{SR} = 8.3 × 10^5 M^{-1} s^{-1} for dilute aqueous solution shown as the dotted line. Adjusted values of k_{SR} in the PRAD model were used to calculate the maximum HO_2 loss rate shown in Supplementary Figure 5b with the same symbol colors as in Supplementary Figure 6a and Fig. 2b in the main text. This strong variation of the HO_2 loss rate with RH over 4 orders of magnitude was necessary to reproduce results here and in Dou et al.[4] The parameterization of k_{SR} in Dou et al.[4] was based off of Corral Arroyo et al. (2018)[12,13] for a system involving photosensitizers and organic radical scavengers. There, the authors constrained k_{SR} to not exceed 8.3 × 10^5 M^{-1} s^{-1} for dilute aqueous solution[12,13]. These studies have found that when RH decreased, k_{SR}

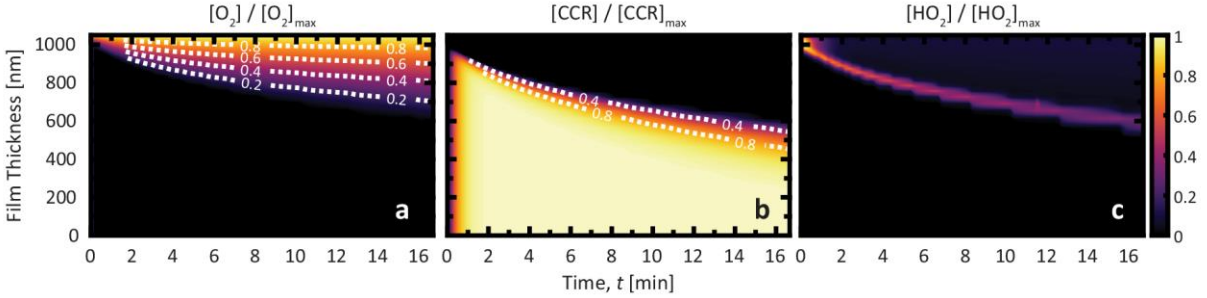

Supplementary Figure 5: Modeled profiles for the coated wall flow tube experiment with an iron(III)-citrate to citric acid mole ratio, M_r = 0.01. **a** Normalized O_2 concentration, $[O_2]$ / $[O_2]_{max}$, **b** normalized carbon centered radical (CCR) concentration, [CCR] / $[CCR]_{max}$, and **c** normalized HO_2 concentration, $[HO_2]$ / $[HO_2]_{max}$, are shown and use the same color scale. The model was run at a relative humidity, RH = 40% and a film thickness of 1.05 µm. Irradiation with UV was switched on at time, t = 0 min. Contours as dotted lines are shown in some instances for clarity. The maximum concentrations are $[O_2]_{max}$ = 6.9 × 10^{-3} mole L^{-1}, $[CCR]_{max}$ = 5.3 × 10^{-2} mole L^{-1} and $[HO_2]_{max}$ = 1.8 × 10^{-6} mole L^{-1}.

potentially was reduced due to a water activity dependent rate coefficient. Yet, it is not clear if k_{SR} must be reduced orders of magnitude lower or higher to achieve model agreement with observations than what is expected in dilute aqueous solution[4,13]. We also note that for O_2, M_r, thickness and light dependent experiments, k_{SR} must be adjusted about 1 - 3 orders of magnitude higher than values determined in Dou et al. (2021)[4] at the same *RH*.

These findings and our results all point toward a production or loss mechanism of HO_2 not included in detail in PRAD. In the condensed phase, the initial attack near the OH group of the intact citric molecules leads to elimination of HO_2. However, most secondary oxidation steps, either further decarboxylation or after an attack of OH radicals, may lead to formation of peroxy radicals, RO_2. Then, RO_2 can be a sink for HO_2. In the model, C5 photochemical degradation leads to CO_2 formation (and thus mass loss), but does not form RO_2. Supplementary Figure 6c shows the HO_2 turnover, -d[HO_2]/d*t*, calculated from the product of k_{SR} from Supplementary Figure 6b and the squared maximum HO_2 concentration, $([HO_2]_{max})^2$. The turnover is independent of *RH* and has a variability much smaller than k_{SR}. If we were to consider the reaction between RO_2 and HO_2, then it is likely that this is slow at low *RH* due to a reacto-diffusion limitation. In other words, RO_2 should be a radical with a diffusion coefficient as low as citric acid and other C_5 compounds. Therefore, the HO_2 sink with RO_2 should be very small when $RH <$ 30%. Adjusted k_{SR} values were greater than 8.3×10^5 M^{-1} s^{-1} when $RH >$ 30%, at which we suspect the additional HO_2 sinks mentioned above become important. This would impact the product generation and the balance between ROS species of the overall photochemical system. We note multi-phase OH chemical

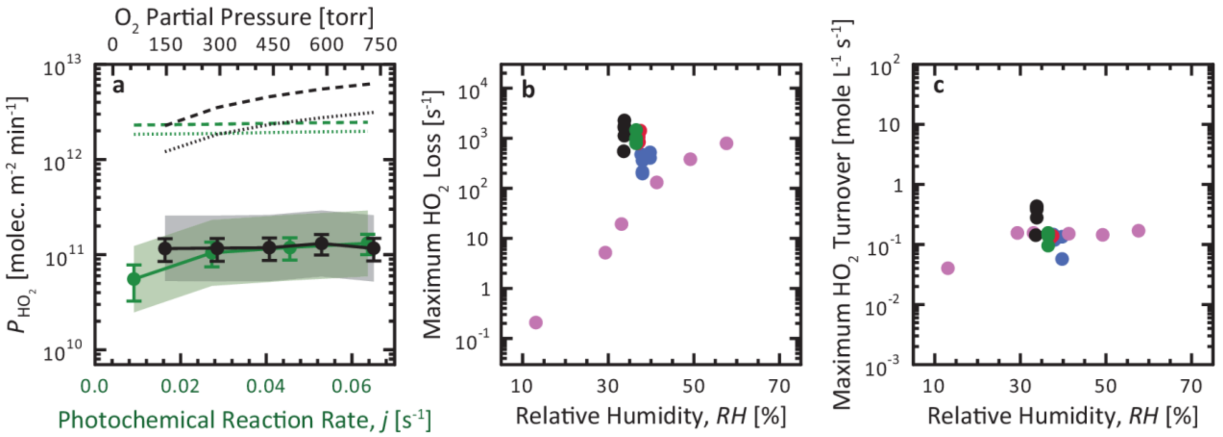

Supplementary Figure 6: HO_2 production, P_{HO_2}, in coated wall flow tube (CWFT) experiments. a Measured and model derived production of HO_2 as a function of light intensity (green) and O_2 partial pressure (black) is shown. Light intensity is translated to the photochemical reaction rate, *j*. Symbols are measured P_{HO_2}, solid lines are model predictions using HO_2 self reaction rate coefficients, k_{SR}, adjusted to match observations and the shading is the model sensitivity to a factor of 5 error in k_{SR}. Dashed and dotted lines were determined from parameterization of k_{SR} as a function of relative humidity, *RH*, or $k_{SR} = 8.3 \times 10^5$ M^{-1} s^{-1} in dilute aqueous solution, respectively[4]. Here, M is molarity in units of mole L^{-1}. **b** The maximum HO_2 loss rate derived for CWFT experiments when *j*, O_2 partial pressure, iron(III)-citrate to citric acid mole ratio, M_r, and thickness was varied as green, black, blue and red symbols, respectively. Loss rates calculated from Dou et al.[4] as a function of *RH* are shown as purple symbols. **c** The maximum HO_2 turnover rate determined from the model. Symbol colors are the same as in panel **a** and **b**.

reaction with citric acid has been a subject of study all in itself with a particular focus on product generation[32]. A full representation of OH and RO_2 chemistry in the condensed phase, however, is beyond the scope of our study. In general, HO_2 and RO_2 chemistry results in the formation of peroxides and O_2, which would go on to react with CCRs to form more HO_2. Therefore, it is likely the total budget of CCRs and ROS would not significantly be affected by not treating this chemistry explicitly.

Concentration Profiles of Radicals and Reactants in Films

Supplementary Figure 7a shows concentration profiles of selected compounds in films. Of particular interest is the model derived profile of HO_2 shown as the green line having 2 local maxima appearing about 20 and 80 nm from the film surface. Near the surface, there was an ample amount of O_2 as seen by the blue curve, indicating that both the O_2 reaction with CCRs and iron(II) citrate caused this first peak in condensed phase HO_2. The second peak of HO_2 concentration is larger and indicates a more rapid production in films due to the reaction of O_2 and CCRs, which were at a much higher concentration present throughout the majority of the 1.8 µm thick film than near the film surface. Notice that O_2 and CCRs (purple curve) completely deplete each other and decay to zero at a depth of ~60 nm from the film surface. This O_2 depletion depth is larger than for STXM/NEXAFS experiments seen in Supplementary Figure 2 and in Supplementary Table 1. This is entirely due to M_r = 1.0 in STXM/NEXAFS experiments being larger than for thickness dependent CWFT experiments where M_r = 0.08. When M_r decreased, the concentration of photochemically produced CCRs also decreased and O_2 penetration increased. ROS concentrations were largely due to H_2O_2 and shown to have been at a maximum concentration near the film surface and about 10% of its maximum value at the bottom of the film. Supplementary Figure 7b shows the absolute concentrations of the ROS constituents, H_2O_2, HO_2, OH and $O_2^{\cdot-}$. Notice that H_2O_2

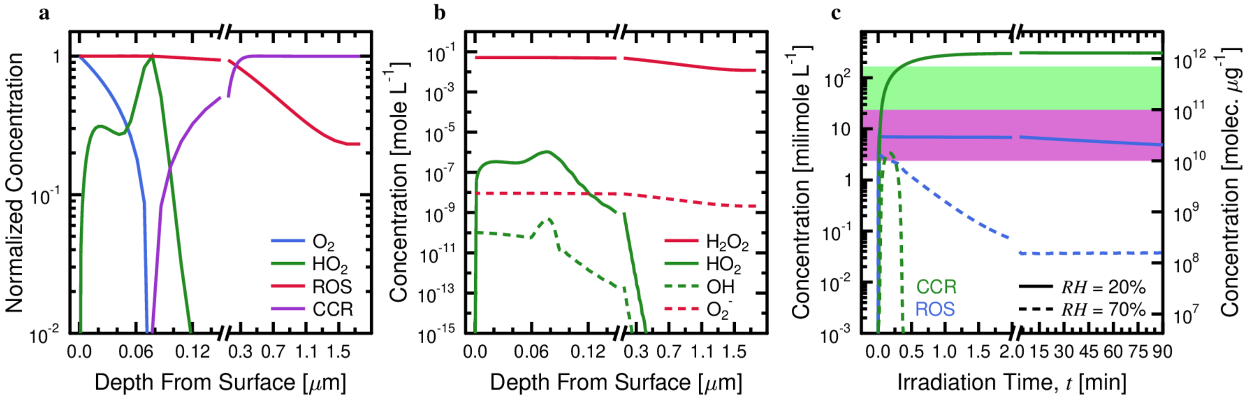

Supplementary Figure 7: Model predicted concentrations in particles and over time. a An example of model derived radial profiles of O_2, HO_2, carbon centered radicals (CCRs) and reactive oxygen species (ROS) normalized to their maximum concentration from the coated wall flow tube experiment with a film thickness of 1.8 µm seen in Fig. 2b of the main text. Normalized concentrations are taken at 15 minutes of UV irradiation. **b** Profiles of the absolute molar concentrations of the ROS constituents in **a**, including H_2O_2, HO_2, OH and O_2^{-}. **c** Average concentration of CCRs and ROS in a particle having a radius of 0.2 µm and iron(III)-citrate to citric acid mole ratio, M_r = 0.05, modelled at a relative humidity, RH = 20% and 70%. The photolysis rate was j = 0.047 s^{-1}, representative for sunlight at the Earth's surface. The right ordinate axis is normalized to aerosol mass using size distributions seen in Supplementary Figure 8a. Green and purple shading are measured concentrations of environmentally persistent free radicals (EPFR) and ROS measured in ambient aerosol particles[5]. Note the abscissa scale break in both panels.

produced at the oxygen rich near surface region diffuses toward the center where the anoxic region is. OH concentration mirrors that of H_2O_2 due to the production and loss solely due to H_2O_2 and Fe^{2+}. This is different from HO_2 and $O_2^{\cdot -}$, which are produced only near the surface. As previously discussed, peroxy radical or hydroperoxides may have existed in films and particles in the bulk, although their reaction with HO_2 or other compounds is not explicitly represented in the PRAD model. Although, we expect an overall good representation of ROS production, since H_2O_2 may represent the actual H_2O_2 concentration, but also overall peroxide abundance. Similarly for OH and alkoxy radicals, when peroxides react with iron(II) species. They would have anyway contributed to the total ROS concentration and we are, therefore, confident in our quantification of ROS production.

Radicals and Reactive Oxygen Species in Aerosol Particles

Multiphase Chemistry and the Production and Release of Radicals

In order for ROS and CCRs to persist, they must first be formed. Multi-phase chemistry between reactive trace gases and particles can cause ROS and CCR production. Some multi-phase chemical reactions follow a reacto-diffusion limitation[18-20] discussed previously, in which oxidants are confined to shallow layers near the particle surface and condensed phase reactant depletion rates depend on diffusion coefficients, which themselves are a function of temperature, T, and relative humidity, RH. For example, 10% of the organic compound shikimic acid was reacted with ozone at RH = 50% over 3 hours where reactions were limited to the top tens of nanometers, but jumped to an 80% degradation at RH = 70%[33,34]. Previously, semisolid organic aerosols reacted with OH from the gas phase exhibited a highly oxidized aerosol particle shell predicted to occur within about 10 nm of an aerosol surface[35]. If production of ROS and CCRs due to multi-phase chemistry (i.e., gas to particle reactions) only occurs in shallow layers, the chance that they are chemically removed by subsequent reactions or physically removed by evaporation is high, thus confounding the link between radical formation and persistence in aerosol particles beyond metal complexation as previously mentioned. Although, oxidants like OH, O_3, NO_3 are always present in the atmosphere and contribute to aerosol aging, it is unlikely that they cause a significant buildup of additional CCRs or ROS in particles, which may have already contained ROS or radicals. We argue that high CCR and ROS concentrations generated by radicals are instead due to photochemically active species such as the Fe^{III}Cit investigated here.

Production and Persistence of Radicals in Glass- and Liquid-Like Aerosol Particles

The PRAD model has been verified here for a wide range of experimental conditions, including particle diameters, d_p = 0.2 - 20 µm, and film depth, l_f = 0.2 - 2 µm, RH = 0 - 60%, a temperature range of T = 20 - 26.5 °C, M_r = 0.01 - 1.0 and light output from 10^{14} - 10^{20} photons cm^{-2} s^{-1} between 300 - 475 nm (for reference, solar actinic radiation between 300 - 550 nm is on the order of 10^{16} photons cm^{-2} s^{-1}). This makes our model an excellent tool to establish the importance of CCRs and ROS persistence in atmospheric aerosol particles. To do this, we first assumed a hypothetical aerosol size distribution shown in Supplementary Figure 8a with a total concentration of ~330 cm^{-3}, a total mass of 4.5 µg m^{-3}, and that only 1 in 100 particles contained iron complexes irrespective of their size having M_r = 0.05. We ran the PRAD model with these parameters for a particle with d_p = 200 nm, T = 20 °C at either RH = 20% and 70%, which is indicative of highly viscous and more liquid-like states, respectively. Using modeled [CCR], [ROS], the total volume of iron-containing particles and the mass of all particles, we calculated the equivalent

mass normalized [CCR] and [ROS] with units of molecules μg^{-1} of aerosol shown in Supplementary Figure 7c.

At RH = 20%, O_2 limits the depletion of CCRs and slows the turnover of ROS. At RH = 70%, production and loss is much more rapid and more CCRs and ROS are certainly produced. In other words, the PRAD model predicted a burst of radicals at high RH in the first minute followed by a period where the radical concentration drops to a steady state for a long time. This phenomenon is similar to a previously observed burst of OH radicals in dilute aqueous solution containing ambient aerosol particles during the first few minutes of UV irradiation[36]. When [CCR] drops, it is likely that a steady state balance also existed between O_2 reaction and their photochemical production from Fe^{III}Cit (which was also continually being cycled with iron(II) oxidized to iron(III) by ROS and O_2). It is likely that a greater production of ROS due to faster photochemical cycling also occurs with a faster O_2 reaction paired with a ROS loss due to evaporation, leading to low quasi-steady state concentration.

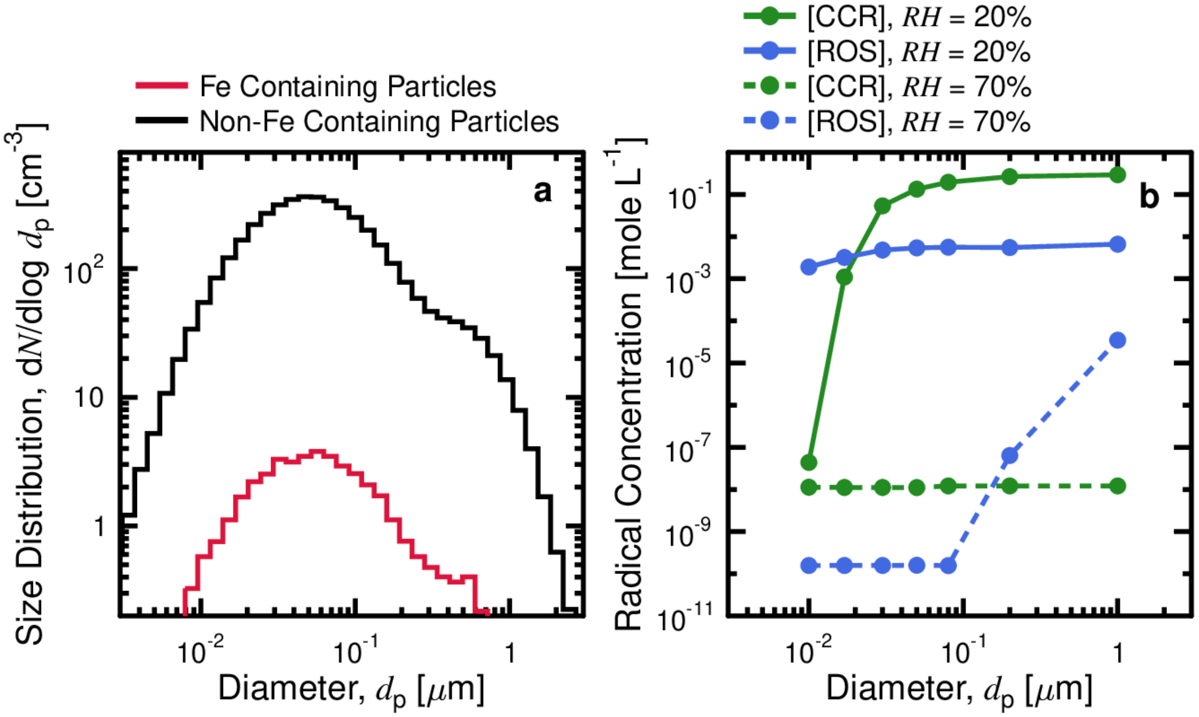

Supplementary Figure 8: Size resolved modelling of carbon centered radicals (CCRs) and reactive oxygen species (ROS) at different relative humidity, *RH*. a Aerosol particle size distribution having a total particle concentration of 330 cm^{-3} and mass of 4.5 $\mu g\ m^{-3}$ for the calculation mass normalized CCRs and ROS shown in Supplementary Figure 7c. Two lognormal modes were used with parameters N_1 = 315 cm^{-3}, μ_1 = 0.05 µm and σ_1 = 0.8 for the first and N_2 = 15 cm^{-3}, μ_2 = 0.5 µm and σ_2 = 0.5 for the second. Iron containing particles were 1% of the total number of particles and have a size distribution seen as red. Non-iron containing particles had a size distribution seen as black. **b** The quasi-steady state concentration of CCRs and ROS, [CCR] and [ROS], respectively, modeled as a function of particle diameter, d_p. The model was run at a temperature, T = 20 °C, using a photochemical reaction rate, j = 0.047 s^{-1}, and an iron(III)-citrate to citric acid mole ratio M_r = 0.05, at RH = 20 and 70% and for d_p = 0.01, 0.017, 0.03, 0.05, 0.08, 0.20 and 1.0 µm. The size distribution, dN / dlog d_p, is a histogram where dN is the particle concentration in a bin and dlog d_p is the diameter bin width on a logarithmic scale.

[CCR] and [ROS] at quasi-steady state with units of M is shown in Supplementary Figure 8b and determined as average values in the final half a model run, i.e., t = 45 - 90 minutes, as a function of particle diameter, d_p = 0.01 - 1.0 μm. We find that as d_p decreases, [CCR] and [ROS] also decreases. When a multiphase reaction follows a reacto-diffusive limitation, the turnover rate is proportional to the surface area to volume and thus proportional to d_p^{-1} for spherical particles[10,18-20,33]. A higher turnover for decreasing d_p implies a greater depletion of CCRs. Therefore, a size dependence of O_2 uptake during photochemical cycling would be expected to some degree in the sub-micrometer particle size range. Although this was the case for CCRs, Supplementary Figure 8b shows that ROS are not as dependent on d_p in highly viscous particles (solid blue symbols). This is due to the fast cycling of ROS and iron oxidation state very near the particle surface. In fact, we have observed that P_{HO_2} changes very little for film thicknesses between 0.2 - 1.8 μm (see Fig. 2b in the main text), which reflects the small change of [CCR] in this same size range.

We estimated that the photochemical processing of highly viscous particles containing trace metal carboxylate complexes should result in concentrations of CCRs and ROS on the order of 10^{12} μg^{-1} and 10^{10} μg^{-1}, respectively. These values are in agreement with reported concentrations of ambient EPFRs and ROS measured with electron paramagnetic resonance (EPR) spectroscopy shown in Supplementary Figure 7c. EPFRs were present at 10^{11} spins μg^{-1}, where “spins” refers to the trapping of EPFRs by the spin trapping agent 5-tert-butoxycarbonyl-5-methyl-1-pyrroline-N-oxide (BMPO) in water[5]. Tong et al. (2016)[37] observed the production of OH and organic radicals when secondary organic aerosol (SOA) mixed with Fe^{2+} decomposed in water. The authors reported that the molar ratio of spin trapped organic radicals to SOA could be as high as 0.4% when the molar ratios of SOA to iron was close to 1:1[37]. At their lowest investigated SOA:Fe ratio of about 20:1, which is similar to our model runs, radicals were produced at 0.05%[37]. Our maximum organic radical concentration of 6×10^{12} μg^{-1} corresponds exactly to 0.1% mole ratio with respect to our particles and is in very close agreement with Tong et al.[37]. The authors later found that organic radical production in water increased by a factor of about 2 when mineral dusts were mixed with SOA at room temperature with, and without, UV light exposure, although this was highly variable and dependent on which dust was present[38].

Radical Release in Lungs After Inhalation

Radical production immediately subsequent to aerosol particles being deposited in water is typically reported in previous studies[5], which is analogous to immediate deposition in the (warm and wet) respiratory tract. However, we predict that photochemical processing of aerosol in the (cold and dry) atmosphere can produce radicals potentially in excess of previous measurements. This would mean that inhaling these atmospheric particles may contribute significantly to radical concentration in lungs and thus result in greater tissue destruction than previously thought. In other words, the total radical production in lungs may be the sum of endogenous and exogenous processes, of which the latter may be due to iron carboxylate photochemistry. In terms of exogenous radical production, consider a human that inhales 10^2 $\mu g\ m^{-3}$ of aerosol in air at a resting rate of 6 L (air) min^{-1}. If the particles contained a CCR concentration of 10^{12} μg^{-1} (see Supplementary Figure 7c and Arangio et al.[5]), then 10^{-2} $\mu M\ hr^{-1}$ radicals would be deposited in the lungs. This is about 10% of the OH production rate ~10^{-1} $\mu M\ hr^{-1}$ determined for ambient particles from urban and rural sites in California, USA deposited in surrogate lung lining fluid[39]. Therefore,

exogenous radical production may be a non-negligible source of lung tissue damage compared with endogenous processes, e.g., further trace metal cycling immediately after inhalation. We suggest that both aerosol exogenous CCR and ROS production be investigated in future studies in addition to endogenic production in lung tissue[39,40].

We argue that photoactive organic aerosol compounds (whether or not iron is present) may lead to radical buildup and adverse health effects when they are inhaled, as is the case with SOA derived from common biogenic and anthropogenic precursors[41]. Organic aerosol mass is roughly a third of the total ambient aerosol mass, which is larger than that of soluble iron. Even if a small fraction of organic aerosol is photoactive[41], it may end up exogenously producing a significant number of radicals. In atmospheric particles, radical persistence would allow their concentration to build up to levels beyond those determined considering well mixed particles, i.e. homogeneous distribution of O_2 and other species. CCRs and ROS preserved or locked in highly viscous particles means their lifetime is long. When particles with high radical concentration are inhaled, they release not only the organic radicals and ROS in the respiratory tract, but also the CCR and ROS precursors, i.e. organic or iron species. Then, they are immediately mixed in the warm and saturated lung environment. Therefore, photochemical cycling before lung deposition can add to the production of endogenous radicals and exacerbate tissue damage.

Radical and Reactive Oxygen Species as a Function of Environmental Parameters.

We estimated [CCR] and [ROS] in aerosol particles using the PRAD model as a function of T, RH, M_r and light intensity in Fig. 3 of the main manuscript and Supplementary Figure 9 to investigate what environmental factors would cause radical build up in aerosol particles. Light intensity was represented by j calculated using the wavelength dependent irradiance at the Earth's surface taken from the online tropospheric ultraviolet and visible (TUV) radiation model, the absorption cross section[15] and the quantum yield[4,42]. A maximum value at a solar zenith angle of 0° was $j = 5.23 \times 10^{-2}$ s^{-1}. The model results shown in Fig. 3 of the main text and Supplementary Figure 9 reveal that when T and RH is low, [CCR] and [ROS] is high. This is entirely due to low diffusion coefficients leading to the buildup of CCR and the reactions involving O_2 to form HO_2 and H_2O_2, which is the most abundant ROS species. At RH > 60%, sufficient molecular transport resulted in O_2 quickly finding a CCR reaction partner to form ROS, the latter of which rapidly reacted or evaporated leading to an overall low concentration over time. Concentrations are more dependent on RH than T in the ranges shown in Fig. 3 of the main text. Photolysis rates at the Earth's surface are sufficient to drive the iron complex system toward a quasi-steady state and thus, the CCR concentration is directly proportional to M_r in Supplementary Figure 9c. The drop in ROS at high M_r can be explained by limiting O_2 and thus ROS in particles, i.e. O_2 and ROS inaccessibility to CCRs produced in the bulk of the particle. It is also due to the high concentration of Fe^{2+}, resulting in a much shorter lifetime of ROS.

In addition, we have given a range of T, RH, M_r and j values from various field studies across the globe indicated as boxes in Fig. 3 of the main text and Supplementary Figure 9 with information specific to aerosol iron content, solubility, speciation or complexation[6-9,11]. Meteorological data, i.e. T and RH, for the duration of each field study or sampling time mentioned below are taken from nearby stations to their

respective sampling sites. In some, field studies lasted over seasons and so a seasonal variation is represented. Determination of *j* was made using the site zenith angle, site latitude and the maximum and minimum solar declination angle. The value of M_r was determined from the moles of metal-organic complex and the moles of other compounds in the aerosol particles. We made three conservative assumptions when deriving M_r which were, i) a fraction of iron is soluble, f_{sol}, ii) a fraction of soluble iron is in complex with photoactive organic ligands, f_x, and iii) a fraction of total particles contain iron, f_p. Many previous studies do not report all values necessary for deriving M_r, which complicates its derivation. Wherever possible, we have used reported values and assumed those, which are not available. For

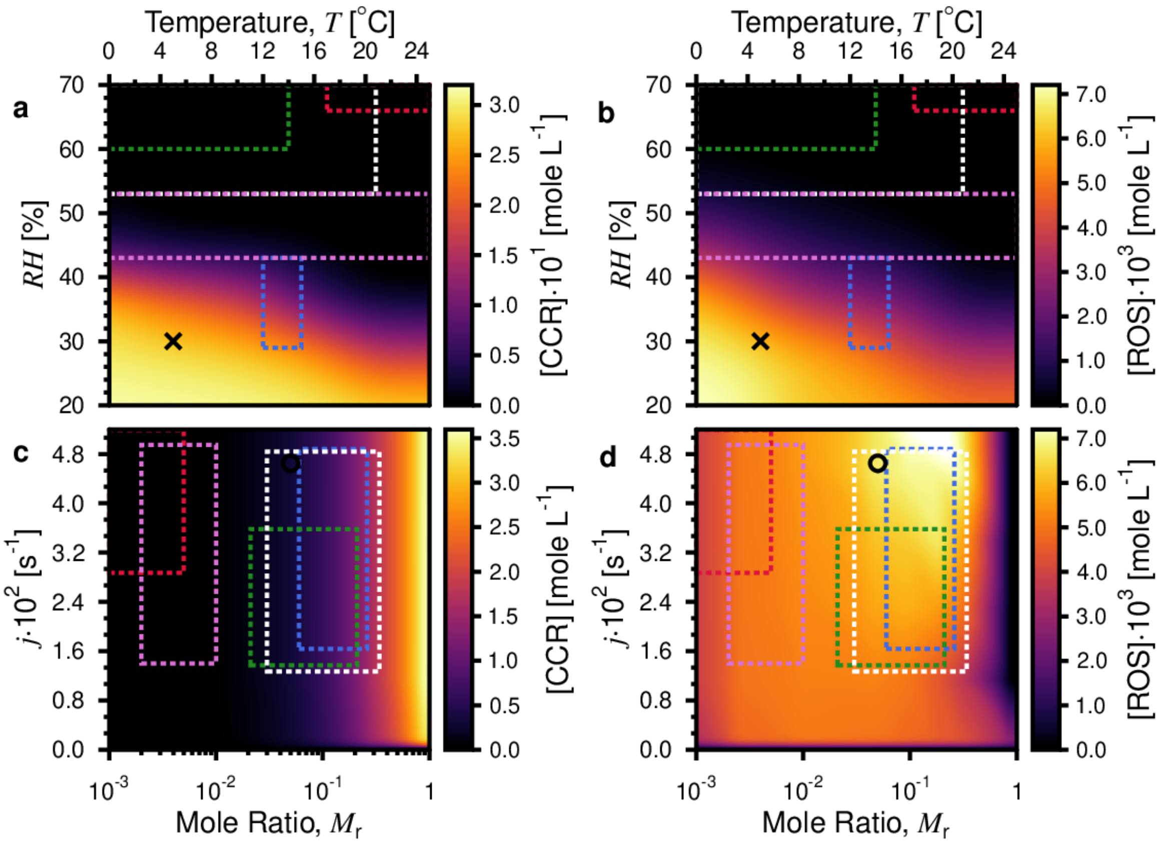

Supplementary Figure 9: Details on the microphysical control for predicting concentrations of carbon centered radicals, [CCR], and reactive oxygen species, [ROS]. a-b Color surface plots of [CCR] and [ROS], respectively, as a function of temperature, *T*, and relative humidity, *RH*. **a** is a reproduction of Fig. 3a in the main text. **c-d** [CCR] and [ROS], respectively, as a function of the iron(III)-citrate to citric acid mole ratio, M_r, and photochemical reaction rate, *j*. **d** is a reproduction of Fig. 3b in the main text. [CCR] is highly dependent on M_r, but relatively independent on *j*, due to the fast photochemical reaction rate and fast cycling pushing almost all iron to the 2+ oxidation state. The quasi-steady state concentration is therefore determined entirely due to M_r and the rate at which O_2 is taken up by particles and reacted. We note that $j = 5.23 \times 10^{-2}$ s^{-1} is the maximum ordinate value and corresponds to the photochemical dissociation rate for iron(III)-citrate with a light intensity at the Earth's surface at 0° zenith. M_r and *j* values in **a-b** are indicated by the circles in **c-d**. *T* and *RH* in **c-d** are indicated by the crosses in **a-b**. Each dashed colored square represents the range of values for the field study in Moffet et al. (2012)[6] in Okinawa Island (red), Takahashi et al. (2011)[7] in China (blue), Tapparo et al. (2020)[8] in Italy (green), Tao and Murphy (2019)[9] in Canada (white) and Hand et al. (2017)[11] in the USA (purple).

simplicity, we have summarized the relationship between concentration and mole ratio from each study mentioned below in Supplementary Table 2 and use the following equation,

$$M_{\mathrm{r}} = M_{\mathrm{r,\,tot\,Fe}}\frac{f_{\mathrm{sol}}f_{x}}{f_{\mathrm{p}}}. \quad (1)$$

The variable, $M_{r,\text{tot Fe}}$, is the mole ratio of iron (soluble and insoluble) out of the total moles in aerosol particles.

We note, that our calculations were not made to determine the exact [ROS] and [CCR] during these field studies, but only to demonstrate under what ambient conditions or typical climate areas may be important for radical persistence as described here. Determination of [ROS] and [CCR] may be highly variable and uncertain due to the variety of aerosol chemical components and their abundance present in the atmosphere. Therefore, the discussion below aims to highlight the importance of radicals in viscous anoxic particles and emphasize the need for their further measurement in the aerosol phase.

Supplementary Table 2: Values for aerosol iron content in field studies. [Fe_{tot}] is the total iron concentration, $M_{r,\text{ tot Fe}}$ is the mole ratio of iron, f_{sol} is the soluble iron fraction, f_x is the fraction of soluble iron in complex with organic ligands, $f_{Fe,\text{ p}}$ is the fraction of particles that contain iron and M_r is the mole ratio of iron organic complexes in aerosol particles.

Location	Notes	[Fe_{tot}] / nanomole m^{-3} (air)	$M_{r,\text{ tot Fe}}$	f_{sol}	f_x	$f_{Fe,\text{ p}}$	M_r
**Southern Great Plains, USA[11]**	**Aerosol composition given. Fe solubility assumed.**	**4.1 - 20.7**	**0.4 - 2.2 × 10^{-2}**	**0.03**	**0.76**	**0.05**	**0.2 - 1.0 × 10^{-2}**
**Aksu, China[7]**	**Aerosol composition and solubility given.**	**0.03 - 1.24 × 10^{3}**	**0.13 - 0.14**	**0.03 - 0.12**	**0.76**	**0.05**	**0.6 - 2.6 × 10^{-1}**
**Urban Areas, Canada[9]**	**Soluble iron and organic concentration given.**	**0.2 - 3.0**	**0.1 - 1.1 × 10^{-1}**	**0.20**	**0.76**	**0.05**	**0.3 - 3.4 × 10^{-1}**
**Po Valley, Italy[8]**	**Aerosol composition and solubility given.**	**1.5 - 14.8**	**0.2 - 2.4 × 10^{-2}**	**0.11**	**0.76**	**0.05**	**0.3 - 4.0 × 10^{-2}**
**Okinawa Island[6,43]**	**Iron mole fraction derived from STXM detection limit.**	**0.1 - 1.5**	**0.2 - 2.3 × 10^{-1}**	**0.03**	**0.76**	**0.05**	**0.5 - 5.0 × 10^{-3}**

In the study by Moffet et al.[6], ambient particles in air originating from a continental and urban source were collected from Okinawa Island, Japan. The authors determined $\beta = 0.66 \pm 0.08$ and that only about 1:20 particles had detectable iron using STXM/NEXAFS. Therefore, for this study and the others below, we have assumed $f_{Fe,p} = 0.05$. The range of T and RH were determined from measurements obtained during the same aerosol sampling times at the Cape Hedo Atmosphere and Aerosol Monitoring Station. The range of j was determined from the year-round range of solar zenith angles. A range of M_r was much less trivial to obtain and required some estimation as follows. In general, the detection limit of STXM/NEXAFS is exactly related to the number of atoms in the X-ray beam. Consider that the atomic ratio for carbon, oxygen and iron at $M_r = 0.05$ is C:O:Fe = 126:147:1. Using a particle density of 1.6 g cm^{-3}, the X-ray attenuation length, h_{atten} = 0.70 µm and 0.68 µm at the pre-edge and post-edge energy ranges for iron (700 - 704 eV and 730 - 735 eV, respectively). This ~2% difference in h_{atten} would be indiscernible in STXM/NEXAFS, however, resonant peak heights at 707.8 and 709.5 eV for identifying oxidation state are typically 10× more absorbing than the edge step[6,10]. Using a typical incident X-ray photon count per pixel

of I_0 = 1000, and a thickness of h = 0.5 µm, the expected transmitted photons, I, and optical density, OD, can be determined from

$$OD = -\ln\frac{I}{I_0} = \frac{1}{h_{\text{atten}}}h. \tag{2}$$

Plugging I_0 = 1000, h = 0.5 µm and h_{atten} = 0.70 µm into Eq (1) above, the transmitted photons at the pre-edge expected to be measured is I_{pre} = 488, which is a pre-edge optical density of OD_{pre} = 0.72. At the post-edge, the transmitted photons is I_{post} = 479, leading to a post-edge optical density of OD_{post} = 0.74 and a difference of

$$\Delta OD = OD_{\text{post}} - OD_{\text{pre}}, \tag{3}$$

or ΔOD = 0.018. When adjusting the resonant peak optical density to 10× this difference, i.e.

$$\Delta OD_{\text{adj}} = 10 \times \Delta OD, \tag{4}$$

or ΔOD_{adj} = 0.18 in this example, the adjusted post-edge optical density is

$$OD_{\text{adj,post}} = OD_{\text{pre}} + \Delta OD_{\text{adj}}, \tag{5}$$

or $OD_{\text{adj,post}}$ = 0.89 and the adjusted transmitted photons is $I_{\text{adj,post}}$ = 409 from Eq (1). The error on the OD, σ_{OD}, is propagated through quadrature as

$$\sigma_{OD} = \sqrt{\frac{1}{I} + \frac{1}{I_0}}. \tag{6}$$

Plugging I_0 in $I_{\text{adj,post}}$ to Eq (5) to obtain the error on $OD_{\text{adj,post}}$, $\sigma_{OD\text{adj,post}}$, and plugging in I_{pre} to obtain the error on OD_{pre}, $\sigma_{OD,\text{pre}}$, we can calculate the error on ΔOD_{adj} as

$$\sigma_{\Delta OD_{\text{adj}}} = \sqrt{{\sigma_{OD_{\text{adj,post}}}}^2 + {\sigma_{OD_{\text{pre}}}}^2}. \tag{7}$$

Finally, iron is detectable in STXM/NEXAFS when ΔOD_{adj} determined from Eq (3) is greater than its uncertainty from Eq (6). In the example above, ΔOD_{adj} = 0.18 ± 0.08 and implies that a particle with M_r = 0.05 would be detectable. This procedure was repeated for a range of M_r and plotted in Supplementary Figure 10. Notice that values of ΔOD_{adj} are greater than its error when M_r > 0.0226, which serves as a detection limit imposed on Moffet et al.[6]. This estimation effectively considers other compounds in ambient particles such as NO_3^- or SO_4^{2-}, however the unknown composition should result in a greater uncertainty in the calculated detection limit. Without further information, we conservatively estimate that the total iron mole fraction can range about an order of magnitude greater than this limit, or between 0.023 - 0.23. Assuming a soluble iron fraction of 0.03, and assuming that 76% of soluble iron is in a

carboxylate complex, M_r in iron containing particles can vary between 0.0005-0.005 as indicated as the width of the red box in Fig. 3 of the main text and Supplementary Figure 9.

In the study of Takahashi et al.[7], iron content and solubility in Asian dust undergoing long range transport was observed at various research stations as part of the Aeolian Dust Experiment on Climate impact (ADEC). At one station in Aksu, China, authors reported an iron weight fraction, wt_{Fe} = 4.0% out of total aerosol mass and a solubility of 4.1% measured from leaching experiments in simulated rainwater at pH = 4.7. If iron was distributed equally in every particle, the soluble weight fraction would be 0.2%. However, we assumed that only 1 out of 20 particles contained iron (as in Moffet et al.[6] above) and effectively concentrating up by a factor of 20 or $wt_{sol,Fe}$ = 3.3%. To derive M_r, we needed to first determine the moles of metal-organic complex, n_{moc} and the moles of other compounds, n, so that $M_r = n_{moc}/n$. To derive n_{moc}, we first used the relation that $wt_{sol,Fe} = m_{sol,Fe} / m_p$, i.e. the mass of soluble iron, $m_{sol,Fe}$, per unit mass of total suspended particles in air, m_p. We assumed that 76% of soluble iron was complexed with organic molecules, $n_{moc} = n_{sol,Fe} f_x$, where $n_{sol,Fe}$ is the moles of soluble iron. Therefore,

$$n_{\mathrm{moc}} = \frac{wt_{\mathrm{sol,Fe}}}{M_{\mathrm{Fe}}} m_{\mathrm{p}} f_{\mathrm{x}}, \quad (8)$$

where M_{Fe} is the molar mass of iron. To derive n, we assumed that the moles of other components in the aerosol are ammonium sulfate, n_{AS}, ammonium nitrate, n_{AN}, and citric acid, n_{CA}, as a surrogate for organic

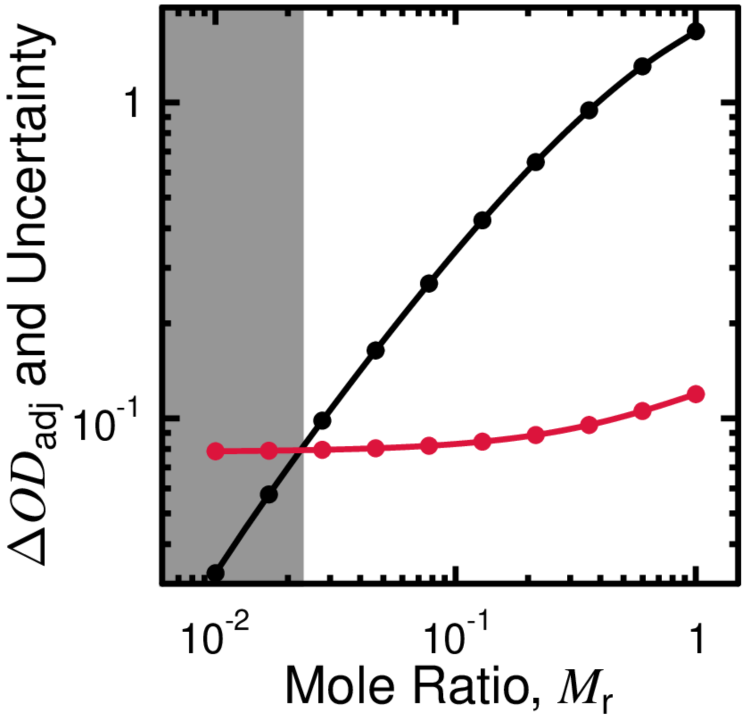

Supplementary Figure 10: Calculation of the iron detection limit in scanning transmission X-ray microscopy coupled to near-edge X-ray absorption fine structure spectroscopy experiments. The difference in optical density between the iron pre L-edge and the resonant peaks, ΔOD_{adj}, is shown in black and its uncertainty is shown in red as a function of the iron(III)-citrate to citric acid mole ratio, M_r. The grey box indicates below detection limit values for $M_r < 0.0226$. See text for more information.

matter such that $n = n_{AS} + n_{AN} + n_{CA}$. We also assumed that the masses of these components are equivalent such that $m_{AS} = m_{AN} = m_{CA}$. Using the relation for the total mass,

$$m_{\mathrm{p}} - m_{\mathrm{moc}} = m_{\mathrm{AS}} + m_{\mathrm{AN}} + m_{\mathrm{CA}}, \tag{9}$$

we derive

$$n = \frac{m_{\mathrm{p}} - m_{\mathrm{moc}}}{3}\left(\frac{1}{M_{\mathrm{AS}}} + \frac{1}{M_{\mathrm{AN}}} + \frac{1}{M_{\mathrm{CA}}}\right) \tag{10}$$

where M_{AS}, M_{AN} and M_{CA}, are the molar masses of ammonium sulfate, ammonium nitrate and citric acid, respectively. We took the metal-organic complex to be iron(III) citrate with a molar mass of M_{FeCit} and thus $m_{moc} = n_{moc}M_{FeCit}$. Then, taking the ratio of Eq (7) and (9) and simplifying yields

$$M_r = \frac{n_{\mathrm{moc}}}{n} = \frac{3wt_{\mathrm{sol,Fe}}f_{\mathrm{x}}/M_{\mathrm{Fe}}}{\left(1 - wt_{\mathrm{sol,Fe}}f_{\mathrm{x}}M_{\mathrm{FeCit}}/M_{\mathrm{Fe}}\right)\left(M_{\mathrm{AS}}{}^{-1} + M_{\mathrm{AN}}{}^{-1} + M_{\mathrm{CA}}{}^{-1}\right)}. \tag{11}$$

Assuming 76% of dissolved iron is complexed and reducing Eq (10) in terms of known constants,

$$M_r = 1.6159wt_{\mathrm{sol,Fe}}\left(1 - 3.3336wt_{\mathrm{sol,Fe}}\right)^{-1}. \tag{12}$$

Plugging in $wt_{sol,Fe}$ = 3.3% gives M_r = 0.06. At another station located in Tsukuba, Japan, M_r = 0.22, which is much higher than in Aksu due to a high iron solubility of about 11.8% and $wt_{sol,Fe}$ = 9.4%. The width of the box for the study of Takahashi et al.[7] was chosen based on these values, i.e. a range of M_r = 0.06 - 0.22. At the eastern Chinese station of Aksu, the climate is sufficiently dry to promote the conditions needed for CCR and ROS buildup.

Tapparo et al.[8] measured the concentration of various metals and organic carboxylate compounds, and their solubility. In addition, inorganic components such as sulfate and nitrate were measured. The authors determined that most iron would be in a carboxylate complex when present in the +3 oxidation state. To estimate M_r of an iron containing particle, we again needed n_{moc} and n. The dataset in Tapparo et al. (2020)[8] is comprehensive and gives the average mass and soluble fraction for 36 elements, 7 organic carboxylates, and 4 major inorganic ions in airborne particles. Using the molar mass of each and considering only the soluble fraction at pH = 4.5[8], we calculated the total dissolved moles per unit volume of air. Using the assumption that 1 in 20 particles contain iron, $n_{moc} = m_{Fe}f_{sol}f_x / (f_pM_{Fe})$. The total moles of species that are not metal-organic complexes are all calculated the same way and leaving major ions, such as sulfate and nitrate, as completely soluble. This results in $n_{moc} = 3.6 \times 10^{-10}$ m^{-3} and $n = 1.4 \times 10^{-7}$ m^{-3}. Considering only the ratio of the soluble fraction of aerosol particles, we calculate M_r = 0.065. Again, we assume that M_r can range about an order of magnitude around this value indicated by the green box in Fig. 3 of the main text and Supplementary Figure 9 having a width of M_r = 0.021 - 0.21. This is equivalent to a concentration of iron carboxylates at about 0.1 - 1 nmol m^{-3} (air). The sampling site in Po Valley tends to be warm and humid, and persistence of radicals is not expected to play a significant role on average. However, the concentration of metals was very similar to other European urban sites and thus may be representative of iron complexes with carboxylates over Europe. Therefore, those cities in

much colder or dryer climates or seasons may then regularly experience such radical buildup in atmospheric particles.

Tao and Murphy[9] measured aerosol composition in urban locations as part of the Canadian national air pollution surveillance (NAPS) program, with a particular focus on oxalate, iron, iron solubility and aerosol pH. The authors report a water soluble iron concentration in ambient aerosol particles from urban Canadian centers between 0.03 - 0.6 nmol m^{-3} (air). Using the same assumptions that 76% is in a carboxylate complex and only 1 in 20 particles contain iron, we estimate that iron carboxylate concentration in single iron containing particles can be between 0.5 - 9 nmol m^{-3} (air). For the purpose of calculating M_r, we take an example value of soluble iron concentration reported as $m_{sol,Fe}$ = 0.45 nmol m^{-3} and the corresponding concentration of oxalate between 1.2 - 2.2 nmol m^{-3} from Tao and Murphy (2019)[9]. Again, we assumed that citric acid was the organic ligand in the aerosol population. Considering only organic and iron species, M_r would be between 0.3 - 0.6, which is quite high due to the fact that the concentration of other species was not included. When considering only organic and iron species in the study of Tapparo et al. (2020)[8] above, M_r is a factor of 45 higher than if other major ions, e.g. ammonium, sulfate and nitrate, were included. Scaling M_r by a factor of 45 for Tao and Murphy (2019)[9] and assuming that only 1 in 20 particles contain iron, we find M_r = 0.12 - 0.27. As another example, we choose a lower value for $m_{sol,Fe}$ = 0.1 nmol m^{-3} in Tao and Murphy (2019)[9] with the corresponding oxalate concentration between 0.2 - 1.2 nmol m^{-3}. When using the same factor to account for other major condensed phase ions, i.e. a factor of 45, and additionally considering that 76% of dissolved iron is complexed, then M_r = 0.03 - 0.34. This value spans about 1 order of magnitude and is used as the width of the white box in Fig. 3 of the main text and Supplementary Figure 9. The continental climate in the urban locations investigated in Tao and Murphy (2019)[9] are characterized with sub-zero temperatures for winter and warm summers with the RH typically >50% year round. Therefore, we would suspect photochemical radical formation only on especially dry days.

Aerosol composition was reported in Hand et al. (2017)[11] from the Interagency Monitoring of Protected Visual Environments (IMRPOVE) network with stations positioned across the USA, with a particular focus on the amount of fine dust (FD) from soil and other common aerosol materials (e.g. organic carbon, light absorbing carbon, ammonium sulfate, ammonium nitrate and sea salt). For the purposes of calculating M_r, we have chosen to use the study of Hand et al. (2017)[11] who reported between the years 2011 - 2014. As an example, we have used reported aerosol component mass fractions for a subset of research stations in South Dakota (BADL1 and WICA1), Kansas (CEBL1, TALL1, SAFO1 and NEBR1) and Nebraska (CRES1). Iron solubility was not reported and so we assumed $n_{sol,Fe} = 0.03n_{Fe}$, where n_{Fe} is the total moles of iron determined from the measured iron mass concentration and M_{Fe}. Again assuming citric acid as a proxy for organic carbon, that 1:20 particles contain iron and that 76% of soluble iron is in a metal-organic complex, we have calculated that M_r = 0.002 - 0.010, which was the range of the standard error of measurements. In Fig. 3b of the main text and Supplementary Figure 9c-d, the width of the purple box represents the range conditions for Hand et al. (2017)[11]. In this region of the USA, the climate is moderately dry and cold such that CCR and ROS persistence could frequently occur.

Viscosity and Diffusion Coefficient Dependence on Mole Ratio

In the PRAD model, diffusion coefficients of all species are parameterized as a function of M_r, where a greater Fe^{III}Cit content in particles and films (or larger M_r) yields smaller values of diffusion coefficients. Typically, diffusion coefficients in an organic matrix are inversely proportional to viscosity, η, e.g., in the Stokes-Einstein relation[44,45]. We have measured η for aqueous solution mixtures of citric acid and Fe^{III}Cit using a bench top rheometer (HAAKE™ Viscotester™ iQ air) as a function of M_r and *RH* to assess whether or not η increases with increasing M_r. Measurements were performed using a procedure to ensure that M_r and weight fraction (of total solute) with water, *wt*, were in equilibrium with *RH*. Aqueous samples deposited in the rheometer were enclosed in a chamber having a flow of humidified air at a controlled *RH*. This allowed for sufficient overpressure to avoid ambient air exposure as well. To validate our approach, we reproduced η as a function of *wt* and *RH* for citric acid and sucrose. Parameterizations of *RH* and *wt* determined from previous studies for citric acid[46] and sucrose[47] are available, and therefore, we prepared two-component aqueous solutions of citric acid or sucrose with *wt* corresponding to the *RH* set in the rheometer chamber. Immediately after a solution was prepared, a sample was deposited in the rheometer chamber with a set volume, and η was measured continuously over time. It was often observed that η increased or decreased over time due to the solution evaporating or taking up water during the measurement. This would also lead to a change in solution volume, which could greatly reduce measurement accuracy. Therefore, we avoided these effects by adjusted the *RH* in the rheometer chamber so that η obtained a value within 10% of what was measured in the first minutes of the experiment. This ensured that the adjusted *RH* was close to the prepared *wt* at equilibrium while obtaining η. This procedure was repeated for various values of *wt* and used for all solutions containing sucrose, citric acid and two mixtures of citric acid and Fe^{III}Cit at M_r = 0.05 and 1.0.

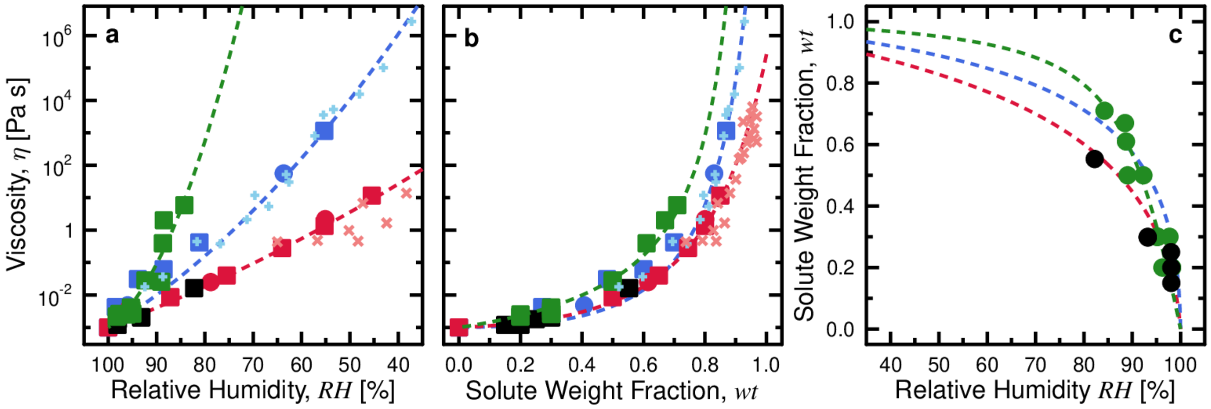

Supplementary Figure 11: Measured viscosity, η, as a function of relative humidity, *RH*, and weight fraction of solutes with water, *wt*. Data for aqueous sucrose (blue), citric acid (red), a mixture with an iron(III)-citrate to citric acid mole ratio, M_r = 0.05 (black) and 1.0 (green) measured in this study are shown as filled symbols. The green dashed lines are parameterizations for the data presented here, where $\log_{10}\eta(RH) = 9.1319 \times 10^{-3} RH^2 + -1.9307 RH + 98.755$ and $a_w(wt) = (1 - wt)(-8.7260 \times 10^{-2} wt^2 + -8.6618 \times 10^{-1} wt + 1)$, where a_w is water activity and 100 × a_w = *RH*. Plus and cross symbols are previous data for sucrose[1] and citric acid[3], respectively. The blue and red dashed lines are parameterizations from previous literature[3].

Supplementary Figure 11 shows η as a function of RH and wt for M_r = 1.0 was much higher than for M_r = 0.05 and citric acid solution without iron. At RH = 84% and M_r = 1.0, the solution had η = 6 Pa s as seen in Supplementary Figure 11a, which is over 2 orders of magnitude higher than for citric acid aqueous solution at the same RH. The same solution at RH = 84% had wt = 0.71 and was 1 order of magnitude higher than a citric acid solution at the same wt shown in Supplementary Figure 11b. For the M_r = 1.0 solution, we have set η as a function of RH and wt equal to each other to determine the relation between these independent variables shown in Supplementary Figure 11c along with a parameterization of $a_w(wt)$ at M_r = 1.0. The uncertainty of η was about ± 10% on the reported value, RH was ± 2% and wt was ± 5% on the reported value. Our procedure of adjusting RH likely introduces additional uncertainty, however, this must not be much more considerable since we have reproduced previous literature and parameterizations of η to well within a factor of 2 and having data with a similar scatter as previous measurements[1,3]. The M_r = 0.05 solution had little effect on η compared to citric acid solutions, in agreement with the parameterization of diffusion coefficients in the PRAD model which change very little for M_r < 0.05[4]. Between M_r = 0.05 and 1.0, diffusion coefficients were predicted to decrease by a factor of 4, which resulted in a good agreement between STXM/NEXAFS (M_r = 1.0) and EDB (M_r = 0.05) experimental results. Further information on the PRAD model and parameterization of diffusion coefficients can be found in Dou et al. (2021)[4].

Supplementary References

1 Power, R. M., Simpson, S. H., Reid, J. P. & Hudson, A. J. The transition from liquid to solid-like behaviour in ultrahigh viscosity aerosol particles. *Chem. Sci.* **4**, 2597-2604, doi:10.1039/C3SC50682G (2013).

2 Lienhard, D. M. *et al.* Retrieving the translational diffusion coefficient of water from experiments on single levitated aerosol droplets. *Phys. Chem. Chem. Phys.* **16**, 16677-16683, doi:10.1039/C4CP01939C (2014).

3 Marshall, F. H. *et al.* Diffusion and reactivity in ultraviscous aerosol and the correlation with particle viscosity. *Chem. Sci.* **7**, 1298-1308, doi:10.1039/C5SC03223G (2016).

4 Dou, J. *et al.* Photochemical degradation of iron(III) citrate/citric acid aerosol quantified with the combination of three complementary experimental techniques and a kinetic process model. *Atmos. Chem. Phys.* **21**, 315-338, doi:10.5194/acp-21-315-2021 (2021).

5 Arangio, A. M., Tong, H., Socorro, J., Pöschl, U. & Shiraiwa, M. Quantification of environmentally persistent free radicals and reactive oxygen species in atmospheric aerosol particles. *Atmos. Chem. Phys.* **16**, 13105-13119, doi:10.5194/acp-16-13105-2016 (2016).

6 Moffet, R. C. *et al.* Iron speciation and mixing in single aerosol particles from the Asian continental outflow. *J. Geophys. Res.-Atmos.* **117**, D07204, doi:10.1029/2011JD016746 (2012).

7 Takahashi, Y., Higashi, M., Furukawa, T. & Mitsunobu, S. Change of iron species and iron solubility in Asian dust during the long-range transport from western China to Japan. *Atmos. Chem. Phys.* **11**, 11237-11252, doi:10.5194/acp-11-11237-2011 (2011).

8 Tapparo, A. *et al.* Formation of metal-organic ligand complexes affects solubility of metals in airborne particles at an urban site in the Po valley. *Chemosphere* **241**, 125025, doi:10.1016/j.chemosphere.2019.125025 (2020).

9 Tao, Y. & Murphy, J. G. The Mechanisms Responsible for the Interactions among Oxalate, pH, and Fe Dissolution in PM2.5. *ACS Earth Space Chem.* **3**, 2259-2265, doi:10.1021/acsearthspacechem.9b00172 (2019).

10 Alpert, P. A. *et al.* Visualizing reaction and diffusion in xanthan gum aerosol particles exposed to ozone. *Phys. Chem. Chem. Phys.* **21**, 20613-20627, doi:10.1039/C9CP03731D (2019).

11 Hand, J. L., Gill, T. E. & Schichtel, B. A. Spatial and seasonal variability in fine mineral dust and coarse aerosol mass at remote sites across the United States. *J. Geophys. Res.-Atmos.* **122**, 3080-3097, doi:10.1002/2016jd026290 (2017).

12 Corral Arroyo, P., Aellig, R., Alpert, P. A., Volkamer, R. & Ammann, M. Halogen activation and radical cycling initiated by imidazole-2-carboxaldehyde photochemistry. *Atmos. Chem. Phys.* **19**, 10817-10828, doi:10.5194/acp-19-10817-2019 (2019).

13 Corral Arroyo, P. *et al.* Particle-Phase Photosensitized Radical Production and Aerosol Aging. *Environ. Sci. Technol.* **52**, 7680-7688, doi:10.1021/acs.est.8b00329 (2018).

14 González Palacios, L. *et al.* Heterogeneous photochemistry of imidazole-2-carboxaldehyde: HO2 radical formation and aerosol growth. *Atmos. Chem. Phys.* **16**, 11823-11836, doi:10.5194/acp-16-11823-2016 (2016).

15 Pozdnyakov, I. P. *et al.* Photophysics of Fe(III)–tartrate and Fe(III)–citrate complexes in aqueous solutions. *Chem. Phys. Lett.* **530**, 45-48, doi:10.1016/j.cplett.2012.01.051 (2012).

16 Berkemeier, T. *et al.* Kinetic regimes and limiting cases of gas uptake and heterogeneous reactions in atmospheric aerosols and clouds: a general classification scheme. *Atmos. Chem. Phys.* **13**, 6663-6686, doi:10.5194/acp-13-6663-2013 (2013).

17 Houle, F. A., Wiegel, A. A. & Wilson, K. R. Predicting Aerosol Reactivity Across Scales: from the Laboratory to the Atmosphere. *Environ. Sci. Technol.* **52**, 13774-13781, doi:10.1021/acs.est.8b04688 (2018).

18 Steimer, S. S., Lampimäki, M., Coz, E., Grzinic, G. & Ammann, M. The influence of physical state on shikimic acid ozonolysis: a case for in situ microspectroscopy. *Atmos. Chem. Phys.* **14**, 10761-10772, doi:10.5194/acp-14-10761-2014 (2014).

19 Hanson, D. R. & Lovejoy, E. R. The reaction of $ClONO_2$ with submicrometer sulfuric acid aerosol. *Science* **267**, 1326-1328, doi:10.1126/science.267.5202.1326 (1995).

20 Worsnop, D. R., Morris, J. W., Shi, Q., Davidovits, P. & Kolb, C. E. A chemical kinetic model for reactive transformations of aerosol particles. *Geophys. Res. Lett.* **29**, 20, 1996, doi:10.1029/2002GL015542 (2002).

21 Renard, P. *et al.* Radical mechanisms of methyl vinyl ketone oligomerization through aqueous phase OH-oxidation: on the paradoxical role of dissolved molecular oxygen. *Atmos. Chem. Phys.* **13**, 6473-6491, doi:10.5194/acp-13-6473-2013 (2013).

22 Herrmann, H. *et al.* Tropospheric Aqueous-Phase Chemistry: Kinetics, Mechanisms, and Its Coupling to a Changing Gas Phase. *Chem. Rev.* **115**, 4259-4334, doi:10.1021/cr500447k (2015).

23 Yang, S. *et al.* Characteristics and seasonal variations of high-molecular-weight oligomers in urban haze aerosols. *Sci. Total Environ.* **746**, 141209, doi:10.1016/j.scitotenv.2020.141209 (2020).

24 Simic, M., Neta, P. & Hayon, E. Pulse radiolysis of aliphatic acids in aqueous solutions. II. Hydroxy and polycarboxylic acids. *J. Phys. Chem.* **73**, 4214-4219, doi:10.1021/j100846a030 (1969).

25 Arangio, A. M. *et al.* Multiphase Chemical Kinetics of OH Radical Uptake by Molecular Organic Markers of Biomass Burning Aerosols: Humidity and Temperature Dependence, Surface Reaction, and Bulk Diffusion. *J. Phys. Chem. A* **119**, 4533-4544, doi:10.1021/jp510489z (2015).

26 Gehling, W. & Dellinger, B. Environmentally Persistent Free Radicals and Their Lifetimes in PM2.5. *Environ. Sci. Technol.* **47**, 8172-8178, doi:10.1021/es401767m (2013).

27 Shiraiwa, M., Selzle, K. & Pöschl, U. Hazardous components and health effects of atmospheric aerosol particles: reactive oxygen species, soot, polycyclic aromatic compounds and allergenic proteins. *Free Radical Res.* **46**, 927-939, doi:10.3109/10715762.2012.663084 (2012).

28 Vejerano, E. P., Rao, G., Khachatryan, L., Cormier, S. A. & Lomnicki, S. Environmentally Persistent Free Radicals: Insights on a New Class of Pollutants. *Environ. Sci. Technol.* **52**, 2468-2481, doi:10.1021/acs.est.7b04439 (2018).

29 Mansano-Weiss, C., Cohen, H. & Meyerstein, D. Reactions of peroxyl radicals with $Fe(H_2O)_6^{2+}$. *J. Inorg. Biochem.* **91**, 199-204, doi:10.1016/S0162-0134(02)00460-9 (2002).

30 Fang, T., Lakey, P. S. J., Rivera-Rios, J. C., Keutsch, F. N. & Shiraiwa, M. Aqueous-Phase Decomposition of Isoprene Hydroxy Hydroperoxide and Hydroxyl Radical Formation by Fenton-like Reactions with Iron Ions. *J. Phys. Chem. A* **124**, 5230-5236, doi:10.1021/acs.jpca.0c02094 (2020).

31 Pozdnyakov, I. P. *et al.* Photochemistry of Fe(III) complex with glyoxalic acid in aqueous solution. *High Energ. Chem.* **43**, 406-409, doi:10.1134/S0018143909050129 (2009).

32 Liu, M. J., Wiegel, A. A., Wilson, K. R. & Houle, F. A. Aerosol Fragmentation Driven by Coupling of Acid–Base and Free-Radical Chemistry in the Heterogeneous Oxidation of Aqueous Citric Acid by OH Radicals. *J. Phys. Chem. A* **121**, 5856-5870, doi:10.1021/acs.jpca.7b04892 (2017).

33 Steimer, S. S. *et al.* Shikimic acid ozonolysis kinetics of the transition from liquid aqueous solution to highly viscous glass. *Phys. Chem. Chem. Phys.* **17**, 31101-31109, doi:10.1039/C5CP04544D (2015).

34 Berkemeier, T. *et al.* Ozone uptake on glassy, semi-solid and liquid organic matter and the role of reactive oxygen intermediates in atmospheric aerosol chemistry. *Phys. Chem. Chem. Phys.* **18**, 12662-12674, doi:10.1039/C6CP00634E (2016).

35 Wiegel, A. A., Liu, M. J., Hinsberg, W. D., Wilson, K. R. & Houle, F. A. Diffusive confinement of free radical intermediates in the OH radical oxidation of semisolid aerosols. *Phys. Chem. Chem. Phys.* **19**, 6814-6830, doi:10.1039/C7CP00696A (2017).

36 Paulson, S. E. *et al.* A light-driven burst of hydroxyl radicals dominates oxidation chemistry in newly activated cloud droplets. *Sci. Adv.* **5**, eaav7689, doi:10.1126/sciadv.aav7689 (2019).

37 Tong, H. *et al.* Hydroxyl radicals from secondary organic aerosol decomposition in water. *Atmos. Chem. Phys.* **16**, 1761-1771, doi:10.5194/acp-16-1761-2016 (2016).

38 Tong, H. *et al.* Reactive oxygen species formed in aqueous mixtures of secondary organic aerosols and mineral dust influencing cloud chemistry and public health in the Anthropocene. *Faraday Discuss.* **200**, 251-270, doi:10.1039/C7FD00023E (2017).

39 Charrier, J. G. & Anastasio, C. Rates of Hydroxyl Radical Production from Transition Metals and Quinones in a Surrogate Lung Fluid. *Environ. Sci. Technol.* **49**, 9317-9325, doi:10.1021/acs.est.5b01606 (2015).

40 Charrier, J. G., McFall, A. S., Richards-Henderson, N. K. & Anastasio, C. Hydrogen Peroxide Formation in a Surrogate Lung Fluid by Transition Metals and Quinones Present in Particulate Matter. *Environ. Sci. Technol.* **48**, 7010-7017, doi:10.1021/es501011w (2014).

41 Laskin, A., Laskin, J. & Nizkorodov, S. A. Chemistry of Atmospheric Brown Carbon. *Chem. Rev.*, doi:10.1021/cr5006167 (2015).

42 Dou, J. *et al.* Carbon Dioxide Diffusivity in Single, Levitated Organic Aerosol Particles. *J. Phys. Chem. Lett.* **10**, 4484-4489, doi:10.1021/acs.jpclett.9b01389 (2019).

43 Furutani, H. *et al.* Single-particle chemical characterization and source apportionment of iron-containing atmospheric aerosols in Asian outflow. *J. Geophys. Res.-Atmos.* **116**, D18204, doi:10.1029/2011jd015867 (2011).

44 Einstein, A. Über die von der molekularkinetischen Theorie der Wärme geforderte Bewegung von in ruhenden Flüssigkeiten suspendierten Teilchen. *Annalen der Physik* **322**, 549-560, doi:10.1002/andp.19053220806 (1905).

45 Sutherland, W. LXXV. A dynamical theory of diffusion for non-electrolytes and the molecular mass of albumin. *The London, Edinburgh, and Dublin Philosophical Magazine and Journal of Science* **9**, 781-785, doi:10.1080/14786440509463331 (1905).
46 Lienhard, D. M. *et al.* Measurements of Thermodynamic and Optical Properties of Selected Aqueous Organic and Organic–Inorganic Mixtures of Atmospheric Relevance. *J. Phys. Chem. A* **116**, 9954-9968, doi:10.1021/jp3055872 (2012).
47 Zobrist, B. *et al.* Ultra-slow water diffusion in aqueous sucrose glasses. *Phys. Chem. Chem. Phys.* **13**, 3514-3526, doi:10.1039/C0CP01273D (2011).
